# Multi-Integration of Labels across Categories for Component Identification (MILCCI)

**Published:** 2026-02-04

**Authors:** Noga Mudrik, Yuxi Chen, Gal Mishne, Adam S. Charles

**Affiliations:** 1 Biomedical Engineering, Kavli NDI, The Mathematical Institute for Data Science, Center for Imaging Science, The Johns Hopkins University, Baltimore, MD; 2 Department of Neuroscience, The Johns Hopkins University, Baltimore, MD; 3 Halıcıoğlu Data Science Institute, UCSD, San Diego, CA.

## Abstract

Many fields collect large-scale temporal data through repeated measurements (‘trials’), where each trial is labeled with a set of metadata variables spanning several categories. For example, a trial in a neuroscience study may be linked to a value from category (a): task difficulty, and category (b): animal choice. A critical challenge in time-series analysis is to understand how these labels are encoded within the multi-trial observations, and disentangle the distinct effect of each label entry across categories. Here, we present MILCCI, a novel data-driven method that i) identifies the interpretable components underlying the data, ii) captures cross-trial variability, and iii) integrates label information to understand each category’s representation within the data. MILCCI extends a sparse per-trial decomposition that leverages label similarities within each category to enable subtle, label-driven cross-trial adjustments in component compositions and to distinguish the contribution of each category. MILCCI also learns each component’s corresponding temporal trace, which evolves over time within each trial and varies flexibly across trials. We demonstrate MILCCI’s performance through both synthetic and real-world examples, including voting patterns, online page view trends, and neuronal recordings.

## 1. Introduction

A key approach to understanding high-dimensional, temporally evolving systems (e.g., the brain) is analyzing time-series data from multiple repeated observations (hereafter *‘trials’*). Each trial is typically labeled with a set of experimental metadata variables. Often, such metadata spans multiple *categories*; for example, each trial in neuronal recordings can be labeled with an attribute from category (a) task difficulty, and from category (b) animal’s choice; each trial in weather measurements can be a time series of temperature over a day, labeled with (a) city, (b) humidity level, and (c) precipitation. We therefore refer to a trial’s *label* as the tuple of its category values, e.g., ‘(easy task, correct choice)’ or ‘(New York, 90% humidity, 1” snow)’. Notably, different trials can have similar or distinct labels. When a category changes between trials (e.g., task difficulty: easy vs. difficult), the corresponding *label entry* changes, while *label entries* corresponding to other categories may remain the same or also change.

Given such multi-trial, multi-label (*‘multi-way’*) data, interpreting how the observations vary across the label space is complicated by the data’s high dimensionality and trial-to-trial variability. A practical approach is to analyze the data in a lower-dimensional latent space (Ma & Zhu, 2013), where label-related structures become easier to interpret. In this space, the activity can be described by a small set of *components*—units that capture the dominant sources of variability across trials and labels. Existing dimensionality reduction methods for analyzing multi-way data often factorize a single, large tensor into such components (Harshman et al., 1970), but they typically overlook trial labels and require constraints on the data structure (e.g., equal-length trials). An alternative is to apply factorizations separately to each trial, which can accommodate varying trial structure but sacrifices information about cross-trial relationships.

Hence, there is a need for new flexible yet interpretable methods to (1) discover the underlying structure within high-dimensional multi-way data, (2) reveal how it captures label information, and (3) disentangle the effect of each category. This, in turn, demands leveraging the trial-to-trial relationships captured by the labels and understanding how these relationships govern the observations.

In this paper, we present MILCCI, a novel method to uncover the underlying structure of multi-way time-series data and disentangle how multi-category labels are embedded within it, both structurally and temporally. Our contributions include:

- We introduce MILCCI, a flexible model that discovers interpretable sparse components underlying multi-way data and reveals how they capture diverse label categories.
- We identify components that capture label-driven variability across trials and track how their activations evolve within individual trials, thereby encompassing the full spectrum of trial-by-trial variability.
- We validate MILCCI on synthetic data, showing it better recovers true components than other methods.
- We demonstrate MILCCI’s ability to uncover interpretable, meaningful patterns in real data, including the discovery of voting trends across US states that match known events, patterns of online activity reflecting language and device, and neural ensembles supporting decision-making in multi-regional recordings.

## 2. Related Work

The naive approach for analyzing multi-way data is to apply dimensionality reduction individually per trial or jointly across all trials (by stacking multiple trials into a single matrix). This can be done, e.g., via linear matrix decomposition such as PCA, ICA (Hyvarinen et al., 2001), NMF (Lee & Seung, 1999)), sparse factorization for improved interpretability (e.g., SPCA (Zou et al., 2006)), or via non-linear embeddings (e.g., t-SNE (Maaten & Hinton, 2008)). However, per-trial analysis overlooks cross-trial relationships, while analyzing all trials with a single mapping ignores trial-to-trial variability in internal structure.

Demixed PCA (dPCA) (Kobak et al., 2016) isolates task-related neural variance into low-dimensional components, however it does not address missing data, different trial durations, and varying trial sampling rates, which hinders alignment across heterogeneous trials. Mudrik et al. (2024) recently introduced a unified cross-trial model that identifies building blocks encoding label information in multi-array data; however, their method handles only a single dimension of label change and thus cannot disentangle effects of multiple categories that change jointly or separately across trials. TDR and its extensions (Mante et al., 2013; Aoi & Pillow, 2018) capture multi-category labels via per-trial scalar reweighting of fixed matrices, but assume cross-trial variability arises only from linear reweighting of fixed temporal signals and are not tailored to capture variability across trials sharing the same label.

Tensor Factorization (TF), e.g., PARAFAC (Harshman et al., 1970) and HOSVD (Lathauwer et al., 2000), goes beyond individual trials by treating trials as an extra data dimension in a multi-dimensional array. However, existing TF methods, including those incorporating Gaussian processes (Tillinghast et al., 2020; Xu et al., 2012; Zhe et al., 2016) or dynamic information (Wang & Zhe, 2022), are not designed to distinguish label-driven variability from other sources of variability and often produce components that are difficult to interpret. SliceTCA (Pellegrino et al., 2024) extends tensor factorization by simultaneously demixing neural, trial, and temporal covariability classes within the same dataset, allowing components to capture structure across different types of neural variability. However, sliceTCA does not incorporate explicit label information and forces a tensor structure on the data. Chen et al. (2015) enable flexibility in cross-trial representations based on meta-data information, but their assumption of component orthogonality prevents the model from capturing correlated or partially overlapping patterns, limiting their expressive power.

Unlike the methods above, MILCCI (1) identifies the structure of multi-trial, multi-label data that vary over sessions, (2) captures trial-to-trial variability, including within repeated measures of the same label, (3) disentangles how each category is encoded in the data.

## 3. Our Component-identification Approach

### Problem formulation:

**Figure 1.**
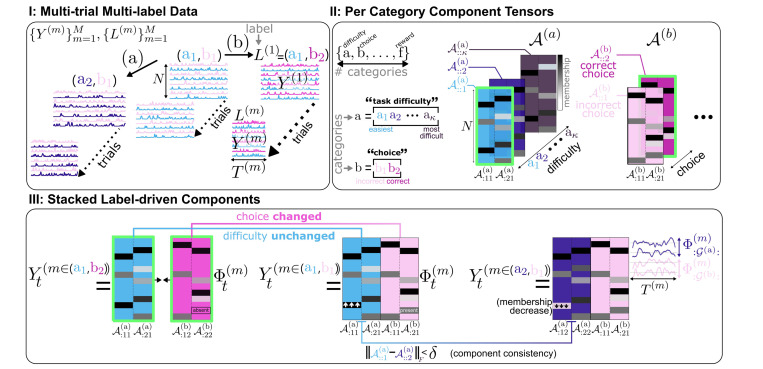
Illustration. **I:** Time-series (e.g., brain recordings) across M trials of varying duration $\left({\left\{T^{\left(m\right)}\right\}}_{m=1}^{M}\right)$. Each trial m is associated with a label $L^{\left(m\right)}$, which is a set of experimental variables spanning different categories (e.g., $L^{\left(m\right)}=\text{(easy task, correct choice)}$). **II:** Each category (k)’s components are represented by a tensor $A^{\left(\text{k}\right)}$, whose i-th variant $\left(A_{::i}^{\left(\text{k}\right)}\right)$ refers to the i-th option of that category (e.g., if the 2-nd option of category (b): correct choice, then $A_{::2}^{\left(\text{b}\right)}$ are correct-choice components). **III:** Each trial m is modeled via a sparse factorization, with its sparse components defined by selecting a variant (layer) from each category’s tensor, based on that trial’s label (green borders, **II**), and then concatenating all selected variants horizontally (green borders, **III**). This forms the loading matrix of that trial. Importantly: 1) trials with identical labels use identical loadings, 2) components can *subtly* adjust their composition under shifts in the respective category values to maintain consistency (e.g., same component under task difficulty 1 vs. 2: ${\left\|A_{::1}^{\left(\text{a}\right)}-A_{::2}^{\left(\text{a}\right)}\right\|}_{F}<\epsilon$), and 3) component temporal traces $\left({\left\{\Phi^{\left(m\right)}\right\}}_{m=1}^{M}\right)$ can vary flexibly across trials.

Let $Y={\left\{Y^{\left(m\right)}\right\}}_{m=1}^{M}$ be a set of M time-series (trials), where each $Y^{\left(m\right)}\in {\mathbb{R}}^{N\times T^{\left(m\right)}}$ represents measurements from N channels across $T^{\left(m\right)}$ time points (Fig. 1, I). We assume that the identities of the N channels are fixed across trials, while trial durations, $T^{\left(m\right)}$, can vary. Each trial $Y^{\left(m\right)}$ is observed under trial-related metadata variables belonging to categories C:={a,b,…,f} (e.g., (a) task difficulty, (b) animal’s choice, etc.), such that |C| is the number of categories. We define the *label* of trial m as the tuple $L^{\left(m\right)}$ containing its |C| metadata variables, such that each *label entry* $L_{i}^{\left(m\right)}$ is the value of the i-th category in that trial (e.g., $L^{\left(m\right)}$ = (task difficulty: easy, choice: correct)). Notably, different trials may exhibit identical labels, partially overlapping labels, or entirely distinct labels. We aim to understand how these labels are encoded within the multi-way observations via their underlying components and traces.

A parsimonious modeling strategy is to model observations $Y^{\left(m\right)}$ in each trial m as being linearly generated by a small set of P core components $A^{\left(m\right)}\in {\mathbb{R}}^{N\times P}$ with corresponding temporal activity $\Phi^{\left(m\right)}\in {\mathbb{R}}^{P\times T^{\left(m\right)}}$, such that $Y^{\left(m\right)}=A^{\left(m\right)}\Phi^{\left(m\right)}+\epsilon$, with ϵ representing e.g., *i.i.d.* Gaussian noise. Particularly, traditional approaches model the data either with per-trial $A^{\left(m\right)}$ reflecting components that change freely over trials, or via a single A shared between trials (via matrix/tensor factorization on stacked trials) such that components are identical across all trials. Consequently, they cannot capture components that *subtly* adjust their composition under label changes across trials.

For example, consider a brain network (i.e., a neuronal ‘component’) that recruits additional neurons during a hard task but not during an easy task. Methods that linearly scale fixed components, if applied independently to trials across task difficulties, would fail to recognize the network as identical across these conditions. Alternatively, if applied to all trials stacked, they would force identical component structures for both easy and difficult tasks, which would misrepresent the additional recruited neurons and would also distort the corresponding traces tied to the components.

Hence, there is a need for methods capable of identifying consistent components underlying multi-way data, understanding how they adapt based on label changes, and capturing their temporal-trace evolution within and across trials.

### 3.1. Our Model:

#### Component Decomposition Approach:

We first assume that to capture category-specific information, each component in A corresponds to a single category (k). Thus, A is composed as a set of |C| category-specific component matrices, ${\left\{A^{\left(\text{k}\right)}\right\}}_{(\text{k})\in C}$, where each $A^{\left(\text{k}\right)}\in {\mathbb{R}}^{N\times p^{\left(\text{k}\right)}}$ consists of $p^{\left(\text{k}\right)}$ components associated with category (k)∈C, such that $\sum_{(\text{k})\in C}p^{\left(\text{k}\right)}=P$. Each entry $A_{nj}^{\left(\text{k}\right)}$ captures channel n’s membership in the j-th component of category (k) (e.g., the extent to which neuron n participates in that neuronal ensemble), and $A_{nj}^{\left(\text{k}\right)}=0$ indicates non-membership (Fig 1, II). We assume that component memberships are sparse, namely each channel n belongs to *only a few* components. Hence, we place a Laplace prior on each entry: $A_{nj}^{\left(\text{k}\right)}∼\text{Laplace}\left(0,\frac{1}{\gamma_{1}}\right)$, where $\gamma_{1}$ is a sparsity-scaling parameter (App. C). Each of the P components exhibits a time-varying trace within each trial (m), collectively represented by the rows of the per-trial traces matrix $\Phi^{\left(m\right)}\in {\mathbb{R}}^{P\times T}$. Then, the observations in each trial are modeled by $Y^{\left(m\right)}\approx \sum_{(\text{k})\in C}A^{\left(\text{k}\right)}\Phi_{G^{\left(\text{k}\right)}}^{\left(m\right)}$, where $G^{\left(\text{k}\right)}$ are the row indices of $A^{\left(\text{k}\right)}$’s traces $\left(\text{length}\left(G^{\left(\text{k}\right)}\right)=p^{\left(\text{k}\right)}\right)$.

#### Extension to Label-dependent Components:

Our full model extends beyond a fixed-component structure; instead, we assume that not only are the components category-specific, the components of each category can exhibit compositional variants by subtly adjusting their membership under label changes. Thus, the representation of each category (k)’s components extends beyond a single matrix to a 3D tensor $A^{\left(\text{k}\right)}\in {\mathbb{R}}^{N\times p^{\left(\text{k}\right)}\times |(\text{k})|}$, where |(k)| is the number of unique label options for that category (e.g., |(k)|=2 for a binary choice vs. |(k)|=κ options for task difficulty, Fig. 1, II). The i-th component variant of category (k) is the i-th slice along the third mode of the component tensor $A^{\left(\text{k}\right)}$ and is denoted by $A_{::i}^{\left(\text{k}\right)}$ (Fig. 1, II).

Thereby, for any trial m with label $L^{\left(m\right)}$, category (k) contributes the component variant $A_{::\arg\left(L_{\left(\text{k}\right)}^{\left(m\right)}\right)}^{\left(\text{k}\right)}$, where $\arg\left(L_{\left(\text{k}\right)}^{\left(m\right)}\right)\in {\mathbb{Z}}_{\geq 0}$ maps a label value to its corresponding variant index within that category (e.g., arg(choice: correct) = 2, as ‘correct’ is the 2nd option in the choice category).

While the variants of each component need not be identical, we assume they are structurally similar, with their similarity level proportional to the similarity of their label values. Thus, for distinct category options ${\left(\text{k}\right)}_{i^{\prime }}\neq {\left(\text{k}\right)}_{i}$ within category (k), we assume that ${\left\|A_{::i^{\prime }}^{\left(\text{k}\right)}-A_{::i}^{\left(\text{k}\right)}\right\|}_{F}^{2}<\delta \left({\left(\text{k}\right)}_{i},{\left(\text{k}\right)}_{i^{\prime }}\right)$, for some small category-dependent distance δ. Notably, this constraint promotes alignment of the components across variants, forcing the $\left\{A^{\left(\text{k}\right)}\right\}$ tensors to capture meaningful synergies between different label combinations rather than overfit to individual trials, ultimately revealing shared yet adaptable patterns across labels.

The decomposition model is thereby extended to the following label-specific formulation for each trial m:

(1) $$Y^{\left(m\right)}=\sum_{(\text{k})\in C}A_{::\arg\left(L_{\left(k\right)}^{\left(m\right)}\right)}^{\left(\text{k}\right)}\Phi_{G^{\left(k\right)}:}^{\left(m\right)}+\epsilon ,\;\epsilon_{ij}\overset{\text{i.i.d.}}{\sim }N\left(0,\sigma^{2}\right).$$

### 3.2. Model Fitting Procedure:

Learning the component compositions and their traces directly from $\left\{Y^{\left(m\right)}\right\}$ is hindered by the mixing of all labels’ effects across categories. We address this through a three-stage procedure:

- *Stage 1: Pre-computing label similarity graphs.* To accommodate both categorical and ordinal labels, MILCCI pre-computes a label similarity graph $\lambda^{\left(\text{k}\right)}\in {\mathbb{R}}^{\left|(\text{k})|\times |(\text{k})\right|}$ for each category (k) (for details see App. A.2). These graphs can be integrated into *Stage 3* to ensure that compositional adjustments reflect label-to-label distances for ordinal labels.
- *Stage 2: Initialization.* We initialize the components and traces following Appendix A.1.
- *Stage 3: Iterative optimization.* Updating ${\left\{A^{\left(\text{k}\right)}\right\}}_{(\text{k})\in \text{C}}$, ${\left\{\Phi^{\left(m\right)}\right\}}_{m=1}^{M}$ until convergence as detailed below.

#### Inferring ${\left\{A^{\left(k\right)}\right\}}_{(k)\in C}$:

For each unique category (k)’s option, ${\left(\text{k}\right)}_{i}$ (e.g., choice: correct), we infer the i-th variant $A_{::i}^{\left(\text{k}\right)}$ using all trials observed under ${\left(\text{k}\right)}_{i}$ $\left(\tilde{M}=\left\{m\in \{1,\ldots ,M\}|L_{\left(\text{k}\right)}^{\left(m\right)}={\left(\text{k}\right)}_{i}\right\}\right)$, regardless of those trials’ values in other categories (e.g., all trials where choice: correct, regardless of trial difficulty).

Since each trial m is modeled via a decomposition of components from multiple categories (Eq. 1), to learn this variant’s $\left(A_{::i}^{\left(\text{k}\right)}\right)$ structure, we need to separate its contribution from the contributions of the other categories’ components. Hence, for each $m\in \tilde{M}$, we first calculate the residual matrix ${\tilde{Y}}^{\left(m,\text{k}\right)}$, which represents the difference between the observations $Y^{\left(m\right)}$ and the partial reconstruction based on all components excluding $A_{::i}^{\left(\text{k}\right)}$. Specifically: ${\tilde{Y}}^{\left(m,\text{k}\right)}:=Y^{\left(m\right)}-\sum_{\left({\text{k}}^{\prime }\right)\neq (\text{k})}A_{::\arg\left(L_{\left({\text{k}}^{\prime }\right)}^{\left(m\right)}\right)}^{\left({\text{k}}^{\prime }\right)}\Phi_{G^{\left({\text{k}}^{\prime }\right)}:}^{\left(m\right)}$. We then infer $A_{::i}^{\left(\text{k}\right)}$ via LASSO (Tibshirani, 1996):

(2) $${\hat{A}}_{::i}^{\left(\text{k}\right)}=\arg\underset{A_{::i}^{\left(\text{k}\right)}}{\min}{\left\|{\tilde{Y}}^{\left(m,\text{k}\right)}-A_{::i}^{\left(\text{k}\right)}\Phi_{G^{\left(\text{k}\right)}:}^{\left(m\right)}\right\|}_{F}^{2}\;\;+\gamma_{1}{\left\|A_{::i}^{\left(\text{k}\right)}\right\|}_{1,1}+\gamma_{2}\sum_{i^{\prime }\neq i}\lambda_{i^{\prime },i}^{\left(\text{k}\right)}{\left\|A_{::i^{\prime }}^{\left(\text{k}\right)}-A_{::i}^{\left(\text{k}\right)}\right\|}_{F}^{2}$$

where $\lambda_{i^{\prime },i}^{\left(\text{k}\right)}$ promotes similarity among same-category (k) variants (App. A.2), and $\gamma_{1}$, $\gamma_{2}$ are hyperparameters controlling sparsity and variant-to-variant similarity. Notably, the model also supports applying a non-negativity constraint on the components $\left(A_{nji}^{\left(\text{k}\right)}\geq 0\;\forall n,j,i\right)$, useful in applications where positive values are expected.

Collectively, Equation 2 balances data fidelity (1st term), component sparsity (2nd term), and consistency between corresponding components proportional to their label similarity by $\lambda^{\left(\text{k}\right)}$ (3rd term). Each component is then normalized to a fixed sum to avoid scaling ambiguity with $\Phi^{\left(m\right)}$.

#### Updating ${\left\{\Phi^{\left(m\right)}\right\}}_{m=1}^{M}$:

Since traces vary independently of labels across trials (i.e., are unsupervised), they can be learned per trial m using the label-driven loading matrix of realized components in that trial. This loading matrix, notated by $A^{\left(L^{\left(m\right)}\right)}\in {\mathbb{R}}^{N\times P}$, is an auxiliary variable constructed by selecting one slice from each category tensor ${\left\{A^{\left(k\right)}\right\}}_{(k)\in C}$ according to the trial’s label $L^{\left(m\right)}$, and horizontally concatenating these slices (Fig. 1, III). In each trial m=1…M, we can then update $\Phi^{\left(m\right)}$ by:

(3) $$\begin{array}{l} {\hat{\Phi }}^{\left(m\right)}=\arg\underset{\Phi^{\left(m\right)}}{\min}\;\underset{\text{data fidelity}}{\underset{︸}{{\left\|Y^{\left(\text{m}\right)}-A^{\left(L^{\left(m\right)}\right)}\Phi^{\left(m\right)}\right\|}_{F}^{2}}}\\ +\gamma_{3}\underset{\text{temporal smoothness}}{\underset{︸}{\sum_{t=1}^{T^{\left(m\right)}}{\left\|\Phi_{:,t}^{\left(m\right)}-\Phi_{:,t-1}^{\left(m\right)}\right\|}_{2}^{2}}}+\gamma_{4}\underset{\text{within-trial trace decorrelation}}{\underset{︸}{{\left\|\left(C⊙\left(1-I_{P}\right)\right)⊙D\right\|}_{1,1}}}, \end{array}$$

where $\gamma_{3}$, $\gamma_{4}$ are hyperparameters, ⊙ is element-wise multiplication, $C:=\mathrm{Gram}\left(\Phi^{\left(m\right)}\right)$, and $D\in {\mathbb{R}}^{p\times p}$ is for normalization $\left(D_{j,j^{\prime }}:={\left\|\Phi_{:j}^{\left(m\right)}\right\|}_{2}^{-1}{\left\|\Phi_{:j^{\prime }}^{\left(m\right)}\right\|}_{2}^{-1}\right)$. Eq. 3 overall promotes data fidelity (1st term), encourages smoothness (2nd term), and penalizes correlations between traces of different components (3rd term). See algorithm, notations, and illustration in Alg.1, Tab.1, and Fig. 1.

## 4. Experiments

We validate MILCCI on synthetic data and also demonstrate its effectiveness on four real-world datasets.

### MILCCI Recovers True Components from Synthetic Data:

**Figure 2.**
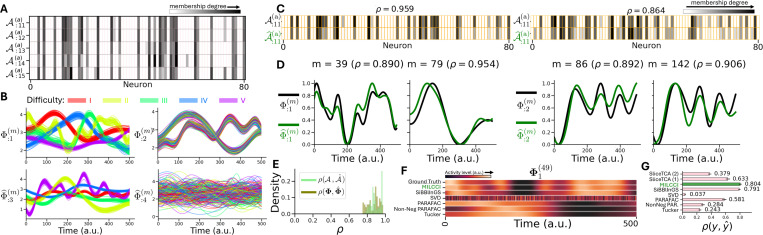
MILCCI Recovers True Representations in Synthetic Data. **A-B:** Generated synthetic data (full data in Fig. 6). Ground-truth components (examples in panel **A**) vary slightly across labels but remain fixed across same-label trials (rows). Ground-truth traces vary across trials (**B**, colored by difficulty). **C-D**: Identified vs. ground-truth components (**C**) and time-traces (**D**) for random trials. **E**: Histogram of correlations between identified components and traces vs. their true counterparts. **F-G**: Comparison of MILCCI to other methods (limited to the same 4-component dimension) based on traces (random trial, **F**) and reconstruction performance (**G**, baselines details in Sec. H).

We generated synthetic data arising from P=4 sparse components with time-varying traces (T=500 time points; M=250 trials). We defined two categories: (a) ‘task difficulty’ (5 options), and (b) ‘choice’ (2 options), such that $p^{\left(\text{a}\right)}=p^{\left(\text{b}\right)}=2$ ensembles adjust with changes in (a) and (b) (Fig. 6B). Each trace was generated as a Gaussian process with parameters varying across components and trials: some reflect task difficulty, some choice, and some vary each trial (Fig. 2, B, Fig. 6, A, App. D). We ran MILCCI on this data, comparing to (1) Tucker (Tucker, 1966), (2) PARAFAC (Harshman et al., 1970), (3) non-negative PARAFAC (Shashua & Hazan, 2005), (4) SVD, (5) SiBBlInGS (Mudrik et al., 2024), and (6) sliceTCA (Pellegrino et al., 2024); all using the same P=4 components (Fig. 2, F,G, Sec.H). Since TF is invariant to component permutation, we used linear sum assignment to align the components of each method with the ground truth.

MILCCI recovered the true components (Fig. 2, C) and traces (Fig. 2, D), with high correlations to the ground truth for both (Fig. 2, E). Compared to other methods (Fig. 2, F,G), MILCCI achieved the highest similarity to the ground truth. While SiBBlInGS attains comparable correlation, it produces blurred traces (e.g., Fig. 2, F) and lacks MILCCI’s interpretability in distinguishing the contribution of each category.

### MILCCI Reveals State-Level Voting Patterns by Party and Office:

We next tested MILCCI on voting data from (Data & Lab, 2017a;b;c) consisting of voting counts for N=51 US states (including DC) across T=23 years (sampled every 2 or 4 years) for different parties (Democrat, Republican, Libertarian; Tab. 3) and offices (Presidency, Senate, House), such that each $Y^{\left(m\right)}\in {\mathbb{R}}^{51\times 23}$ represents the voting counts of all states over years. We first preprocessed the data, including handling missing values due to differing election schedules across states (Fig. 8, App. E).

**Figure 3.**
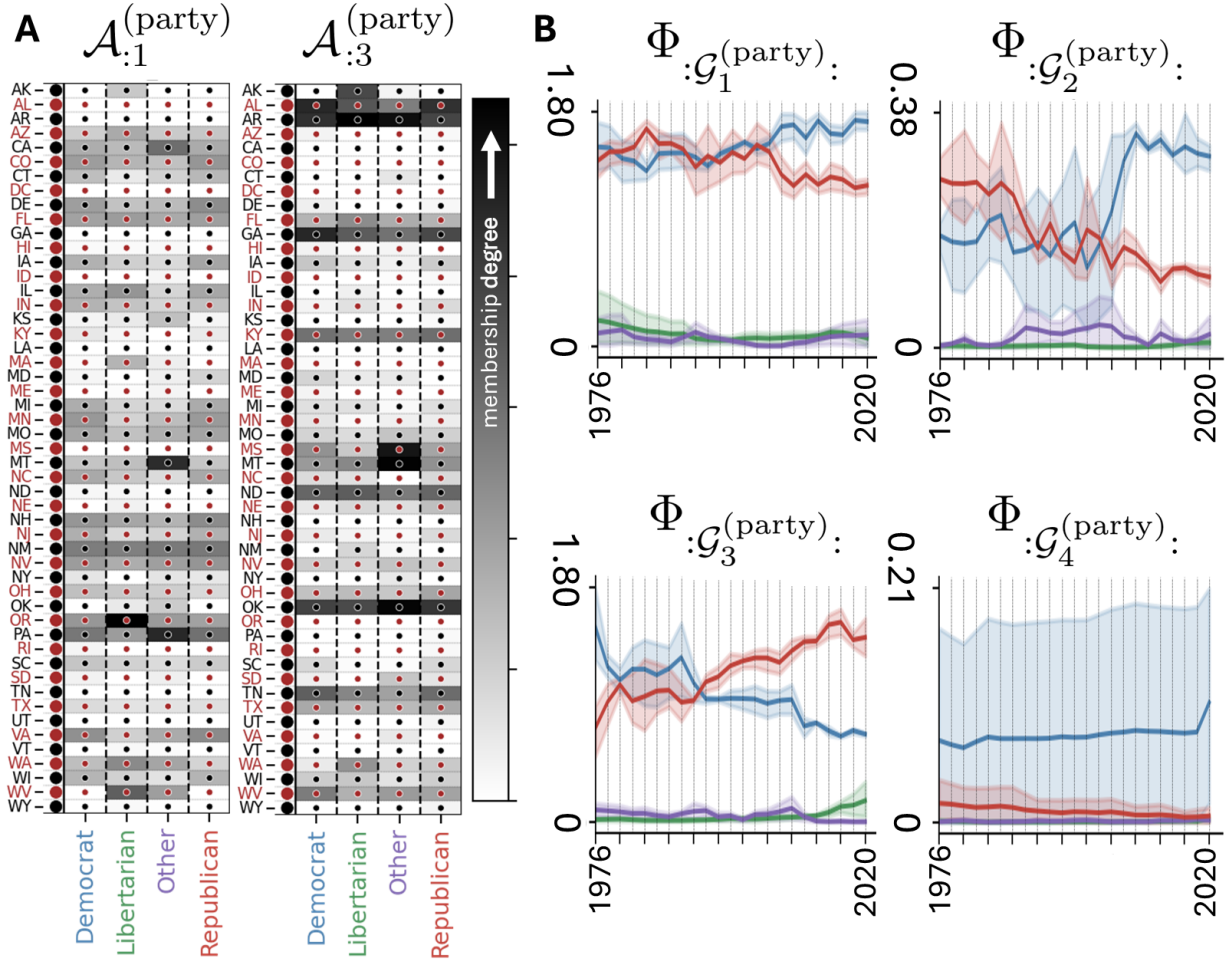
**Voting Results:** Identified example components (**A**) and traces (**B**, Mean and 80% confidence interval). See full in Fig. 8.

We applied MILCCI using P=8 components, with categories (a) party, and (b) office ($p^{\left(\text{a}\right)}=p^{\left(\text{b}\right)}=4$ each). MILCCI discovers components capturing state-specific voting patterns that vary by office, party, and time. For example, component $A_{:1:}^{\text{(party)}}$ (Fig. 3, B.1) highlights Montana (MT) and Pennsylvania (PA) having increased membership in the ‘Other’ category, primarily driven by the Independent Party (Fig. 16, right) and the Constitution Party (Fig. 16, left), respectively. This aligns with MT’s historical emphasis on individualism (Kitayama et al., 2010) and PA hosting the Constitution Party headquarters. The same component identifies Oregon’s increased Libertarian membership, matching the 2001 law that eased ballot access for minor parties (Oregon Political Party Manual) and reflecting that the Libertarian Party of Oregon was among the earliest state branches. Notably, its trace ($\Phi_{:G_{1}^{\text{(party)}}}$, Fig. 3, B.1, top-left) shows overlapping Democrat–Republican activations diverging ~2004, with Democrat activity rising and Republican activity decreasing, reflecting long-term partisan realignment driven by national political shifts of that period (e.g., Iraq War, 2003).

In component $A_{:3:}^{\text{(party)}}$ (Fig. 3, B.1, right), MILCCI groups AK, OK, AL, AZ, MS, MT together. This grouping matches the legislative similarities between these states, e.g., strict voter ID laws (National Conference of State Legislatures, 2025), demonstrating MILCCI’s effectiveness in recovering underlying trends directly from observations. Temporally, its trace $\Phi_{:G_{3}^{(\text{party)}}}$ (Fig. 3, B, bottom-left) shows opposing trends between Democrat and Republican activations, with Republican activation rising, and Libertarian activity emerging around 2016. This trend reflects a rise in Republican votes, a decline in Democratic votes, and a possible shift of some Democratic support toward the Libertarian party in these states. These and other patterns identified by MILCCI (App. E.2) demonstrate its ability to uncover state–party–office–dependent patterns. We further validated (App. E.3) that MILCCI’s discovered voting components capture genuine structures, as individual states show meaningful contributions to reconstruction (Fig. 9, A-C), and the model significantly outperforms permuted null models (*p*<0.001). Additional voting insights are in App. E.2.

Notably, components from other TF methods (Fig. 10, 13) are dense (PARAFAC), include negative values (SVD, PARAFAC, Tucker), or, in SiBBlInGS, fail to disentangle party from office, which hinder interpretability (Fig. 12). Moreover, due to their restrictive tensor structure, PARAFAC and Tucker do not capture compositional adjustments and cannot flexibly vary their traces to capture trial-to-trial temporal variability.

### MILCCI Finds Wikipedia Page Clusters Across Devices and Languages:

**Figure 4.**
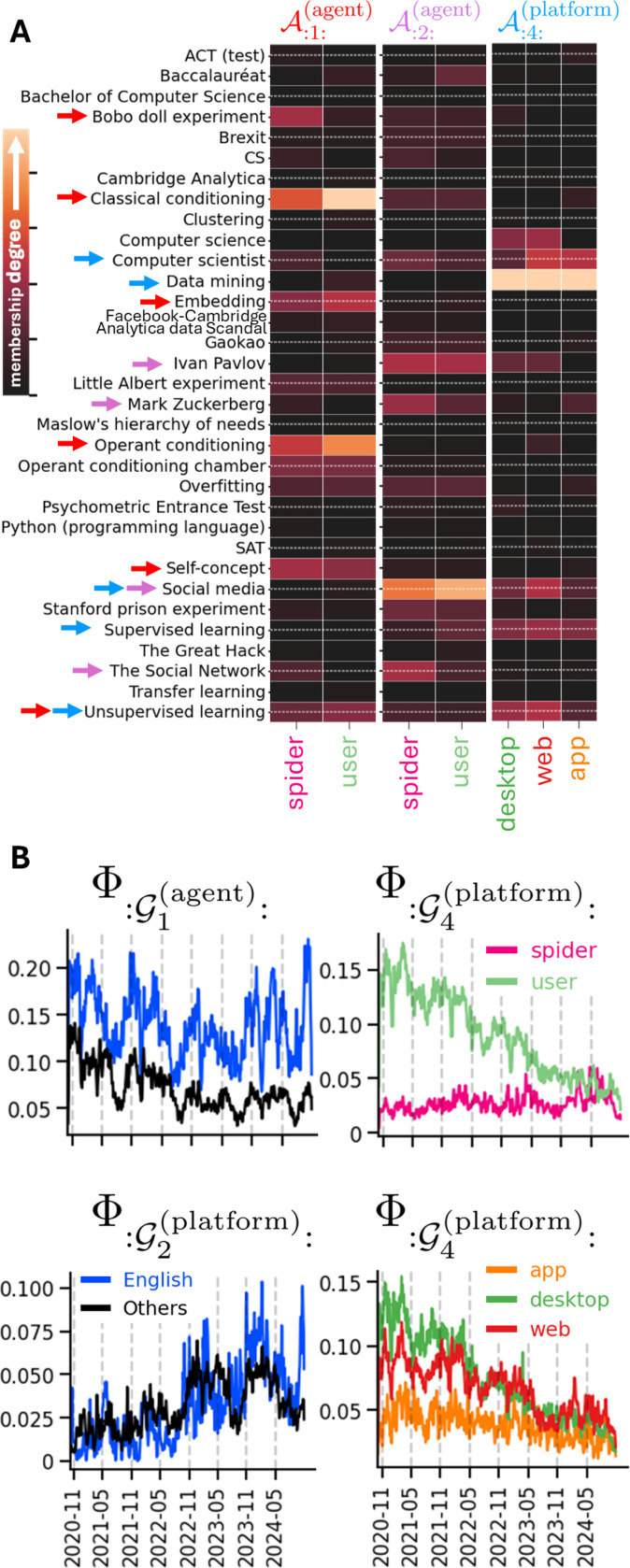
Identified Wiki page-view example components (**A**) and traces averaged by different categories (**B**).

Next, we extracted Wikipedia pages (Wikipages) Pageview counts (Meta, 2022) (Oct. 20’–24’, T=1482 days) for N=32 random Wikipages from different fields. We tracked views under 3 conditions: (a) agent: user/spider (i.e., web crawler), (b) platform: desktop/web/app, and (c) language: en/ar/es/fr/he/hi/zh. Each $Y^{\left(m\right)}\in {\mathbb{R}}^{N\times T}$ contains daily Pageview counts for all 32 Wikipages over time under e.g. (English, desktop, user). We thus seek to identify components capturing Wikipages with similar interest patterns and to understand how these patterns vary across these different conditions (data and pre-processing details in App. F.1). We ran MILCCI on the data with $p^{\left(\text{k}\right)}=4$ components per category, and identified components that cluster related Wikipages together and vary across categories (Fig. 4, A), with some pages (e.g., unsupervised learning) appearing in more than one component, emphasizing MILCCI’s ability to capture multi-meaning terms.

Particularly, we identified, e.g., $A_{:1:}^{\text{(agent)}}$ grouping Learning Theory (in Psychology) related Wikipages (Fig. 4, A, red arrows, list in App. F.2); $A_{:2:}^{\text{(agent)}}$ grouping social-media pages (purple arrows); and component $A_{:4:}^{\text{(platform)}}$ grouping computer science basics (blue arrows). Interesting patterns emerge when exploring how components composition adjust to e.g., user↔spider and desktop↔web↔app.

For $A_{:1;}^{\text{(agent)}}$ (Learning theory in Psychology), MILCCI finds small differences across agents (spider vs. user, Fig. 4, A, left). For instance, the Bobo Doll Experiment’s Wikipage (a psychological experiment on social learning theory) appears under ‘spider’ but not ‘user’. This is consistent with it being less familiar to the average person than other psychology terms in the cluster, while spiders are linked to it through the actual Wikipedia links connecting this page to other related terms. Accordingly, other Wikipages, like classical and operant conditioning, which are foundations in psychology, show higher membership magnitudes in ‘user’. ‘Unsupervised learning’ and ‘embedding’ also show small membership in this component, higher in ‘user’ than ‘spider’. Interestingly, these actual Wikipages refer to CS terms (not psychology), but since they also carry meaning in psychological learning, the higher ‘user’ membership compared to ‘spider’ matches human behavior: users enter the page but leave upon realizing the term refers to a different field, whereas spiders follow predictable navigation patterns. These findings highlight MILCCI’s ability to reveal distinct human vs. spider behaviors within the same component, and also underscore the importance of allowing compositional adjustments to capture subtle component adaptations. This component’s trace ($\Phi_{:G_{1}^{\text{(agent)}};}$, Fig. 4, B top-left) captures its fluctuations and higher activation in English compared to other languages. This aligns with professional terms being more elaborate in English, often using jargon not fully defined/used in other languages, and with non-English native speakers possibly preferring to read professional material in English (Miquel-Ribé & Laniado, 2018).

The social-media component $A_{:2:}^{\text{(agent)}}$ shows small structural adjustments user↔spider: ‘Mark Zuckerberg’ is higher in ‘spider’, while ‘social media’ is higher in ‘user’, possibly reflecting reduced human interest in figures versus common terms like ‘social media’. Its trace $\left(\Phi_{:G_{2}^{\text{(agent)}}:}\right)$ rises until a peak in Mar. 2024 in both English and non-English (Fig. 4 B, bottom-left), matching the general increase in social media popularity over the years and possibly related to, e.g., Florida’s Social Media Ban in Mar. 2024, which is mentioned on the corresponding ‘social media’ Wikipage captured by this component.

$A_{:4}^{\text{(platform)}}$ (computer science basic terms) captures membership adjustments to platform, with, e.g., computer scientist (a short Wikipage without math/graphs) showing higher membership under app/web compared to under desktop. This contrasts with, e.g., unsupervised learning, whose page is more complex (includes math and graphs) and shows lower membership in the app compared to the other platforms. This may match users’ preference to read ‘easy’ terms on the app and more complex terms on desktop, and shows MILCCI’s ability to reveal interpretable processing differences across platforms. This component’s temporal trace $\left(\Phi_{:G_{4}^{\text{(platform)}}}\right)$ shows higher activity in English (Fig. 18), with access dominated by desktop and mobile web (Fig. 4, B, bottom-right). The temporal trace is mostly driven by users rather than spiders (Fig. 4, B, top-right); the user trace decreases over time, aligning with these terms being basic (‘old’) in CS (compared to newer trends, like LLMs). This highlights MILCCI’s power in discovering platform-specific engagements and behavioral fingerprints. Some more interesting patterns include, e.g., $A_{:2}^{\text{(plattorm)}}$ that captures terms related to Cambridge Analytica; its trace peaks around 2020, mainly in English, and decreases since, aligning with the timeline of this case. See Figs. 18, 21, 20, 22, 23 for comparisons and full traces.

### MILCCI Finds Neural Ensembles underlying Multi-Region Brain Data:

**Figure 5.**
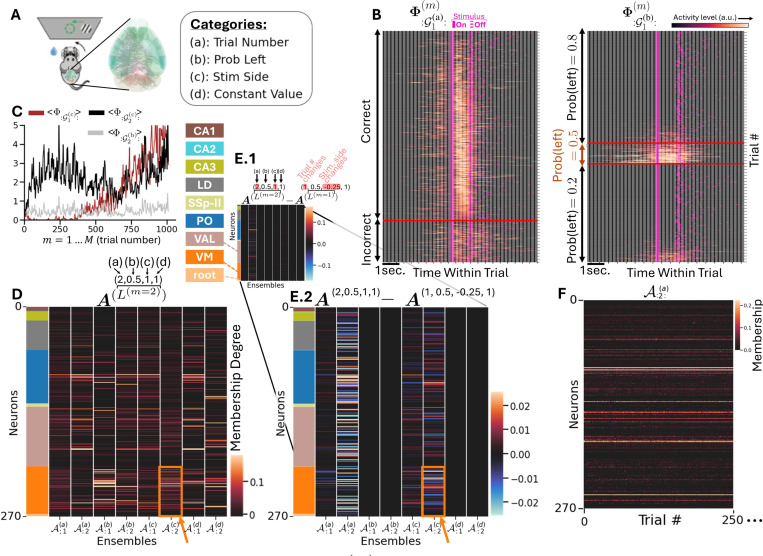
MILCCI identifies meaningful neuronal ensembles in real-world brain data. **A:** Experimental setting (from (Angelaki et al., 2025)). **B:** Component traces (all 1011 trials) sorted horizontally by trial correctness (left panel) and Prob(left) (right panel). **C:** Average within-trial values of exemplary traces reveal varying degrees of temporal drifts over trials. **D:** Ensembles identified (example trial). **E:** Differences in ensemble composition across trials. **F:** Trial-adjusted ensemble compositions over first 250 trials.

We then apply MILCCI to neuronal activity patterns from multi-regional, single-cell-resolution recordings of mice in a decision-making task (Laboratory et al., 2025; Angelaki et al., 2025), App. G). In this experiment, mice reported the location of a visual grating with varying contrast by turning a wheel left or right (Fig. 5, A). We used data from a random session, extracted the available spike time data, and estimated firing rates by applying a Gaussian convolution. We removed inactive neurons and split into M=1011 trials, such that each $Y^{\left(m\right)}\in {\mathbb{R}}^{N\times T}$ with N=270 and T=137 represents the firing rates of neurons over time for trial m (e.g., Fig. 31). We defined $p^{\left(\text{k}\right)}=2$ components (‘neuronal ensembles’) per category: (a) trial number, (b) prior (expected) stimulus side, (c) applied stimulus side, and (d) fixed components across trials. This setup (1) enables distinguishing representational drift (Rule et al., 2019; Driscoll et al., 2022) potentially related to learning or attention, from task variables, and (2) demonstrates MILCCI’s ability to simultaneously support both non-adjusting and adjusting components, across both categorical and ordinal categories.

We found neuronal ensembles selective for diverse task variables (Fig. 5). For example, $\Phi_{:G_{1}^{\left(a\right)}}^{\left(m\right)}$ is tuned to choice correctness, with activity surging during stimulus presentation (Fig. 5, B left) under *correct* choice only. Interestingly, MILCCI also found an ensemble sensitive to *incorrect* decisions, activated just after stimulus presentation ($\Phi_{:G_{2}^{(\text{b}):}}$, Fig. 32). Notably, these components can provide insight as to how neuronal ensembles integrate stimulus information to support correct decision-making, and to relate these traces to the specific neurons involved (Fig. 34). In another example, $\Phi_{:G_{1}^{\left(b\right)}:}^{\left(m\right)}$ is mostly active during trials with a random-prior (i.e., p(left)=0.5), throughout before, during, and after the stimulus, and is largely inactive in trials where the prior favors one side (Fig. 5, B right). This highlights MILCCI’s effectiveness in suggesting involvement of priors in decision-making, and its capability exposing similarities of similar-condition traces.

Moreover, MILCCI’s ability to isolate components with characteristics that track temporal drifts ($\Phi_{:G_{1}^{\left(c\right)},:}^{\left(m\right)}$, Fig. 5, C brown; 33) reveals how neuronal coding evolves over trials, which can be the result of learning, attention, or adaptation. MILCCI reveals how ensemble compositions capture distinct local and widespread structures across regions (Fig. 5, D; colors on the left mark neurons’ brain regions). For example, $A_{:2:}^{\left(\text{c}\right)}$ captures many neurons in the Ventral Medial nucleus of the thalamus (VM, orange arrow in Fig. 5D), suggesting it may be involved in arousal regulation and motor coordination. We can also identify interesting patterns via the ensemble compositions (Fig. 5, D), and how they change between trials (e.g., between two example trials with differing stimulus sides; Fig. 5, E.1, zoom-in on changes in Fig. 5, E.2). These reveal patterns in ensemble composition adjustments: the first ensemble of $A^{\left(\text{a}\right)}$ (Fig. 5, E.2, left column) adjusts minimally, while the second ensemble exhibits distributed adjustments across areas. In stimulus-adjusting ensembles (columns 5,6), adjustments occur around VM (e.g., $A_{:2:}^{\left(\text{c}\right)}$, Fig. 5), suggesting an adaptive VM composition.

### MILCCI Identifies Arousal- and Frequency-Dependent Neuronal Ensembles:

Finally, in App. J, we show that MILCCI identifies components capturing arousal and stimulus frequency, with robustness to hyperparameters.

## 5. Discussion, Limitations, and Future Steps

We presented MILCCI, a data-driven method for analyzing multi-trial, multi-label time series. MILCCI identifies interpretable components underlying the data, captures cross-trial relationships, and integrates label information, while accounting for trial-to-trial variability beyond label effects. MILCCI allows components to adjust their composition with label changes, which uncovers similarities that could remain hidden under fixed-component factorizations. Unlike other approaches, MILCCI maintains interpretability while modeling cross-trial variability, integrating labels, and disentangling label-driven from non-label-driven sources of variation. Another strength of MILCCI is its ability to simultaneously handle multiple label types within a single dataset, including categorical, non-continuous ordinal, continuous, and trial-varying labels (e.g., as in the IBL experiment). We validated MILCCI on synthetic data, where it outperformed alternatives, and demonstrated its effectiveness on real-world data: (1) exposing voting patterns aligned with real events; (2) recovering interpretable Wikipedia view components with memberships varying across languages, platforms, and agents; and (3) revealing neuronal ensembles adapting across trials and task variables. Notably, MILCCI’s architecture (low-rank, sparse components) naturally constrains the model to learn patterns that are flexibly reused across trials. Another feature of MILCCI is its modular design, which supports substitution of our current MSE metric (reflecting normal distribution assumptions) with alternative application-specific cost metrics in the future. Notably, while here we focused on time series for clarity, MILCCI is applicable to modalities beyond temporal data.

One potential concern is the scaling ambiguity between components and traces, which MILCCI addresses by normalizing components after each iteration while allowing traces to flexibly vary across trials to capture trial-specific amplitudes. Although component dimension per category is a hyperparameter, MILCCI naturally handles this, as sparsity drives redundant components to zero. Future directions include extending the linear assumption, central to interpretability, to non-linear structures (e.g., via kernelization); parallel optimization or batch processing for large datasets; or dynamic priors over component time evolution (e.g., App. I, J).

## Supplementary Material

Supplement 1

## Figures and Tables

**Table 1.** Key notations.

Notation	Description
m	Trial #
(k)∈C	Category
$L^{\left(m\right)}=\left(L_{\left(\text{a}\right)}^{\left(m\right)},L_{\left(\text{b}\right)}^{\left(m\right)},\ldots \right)$	Label of trial m
$Y^{\left(m\right)}\in {\mathbb{R}}^{N\times T^{\left(m\right)}}$	Trial m’s observations
$\Phi^{\left(m\right)}\in {\mathbb{R}}^{P\times T^{\left(m\right)}}$	Trial m’s traces
$G^{\left(\text{k}\right)}$	Indices of category (k)’s traces
$A^{\left(\text{k}\right)}\in {\mathbb{R}}^{N\times p^{\left(\text{k}\right)}\times |(\text{k})|}$	Category (k)’s components
$A^{\left(L^{\left(m\right)}\right)}\in {\mathbb{R}}^{N\times P}$	Trial m’s components

## Impact Statement

This work advances machine learning methods for analyzing multi-view data across diverse domains. We do not anticipate negative societal consequences from this methodological contribution.
