# Supplementary material for: Multi-Integration of Labels across Categories for Component Identification (MILCCI)
Source: ArXiv. 2026 Feb 4:arXiv:2602.04270v1. Preprint. [Version 1] (PMC12889858)
Supplement: Supplement 1 [file NIHPP2602.04270v1-supplement-1.pdf]

Appendix

Contents

A Additional Fitting Details: **14**
A.1 Initialization: 14
A.2 Details about state similarity graph calculation: 14
A.3 About sparsity and consistency hyperparameters 14

B Running Details **15**
B.1 Data and Code Availability 15
B.2 Versions 15

C Elastic Net Prior of Components **15**

D Additional Information—Synthetic Experiment **15**

E Additional Information—Voting Experiment **16**
E.1 Voting Data Pre-Processing 16
E.2 Additional Findings—Voting Data 18
E.3 Post-hoc Validation Analysis on Voting Data 20

F Additional Information–Wikipedia Experiment **30**
F.1 Wikipedia Pageview Data Pre-Processing 30
F.2 Clarification on Findings—Wikipedia Data 31

G Additional Information about Neuronal Ensembles Experiment **39**

H Information About Baseline Calculation and Execution **39**
H.1 Comparison to SVD, Tucker, PARAFAC 39
H.2 Comparison to SliceTCA 40
H.3 SiBBlInGS 40

I Alternative Inference of traces via Dynamic Prior **41**
I.1 Linear Dynamical System Prior 41
I.2 Modified Objective and Inference 41
I.3 Initialization 41

J Fourth real-world experiment: MILCCI identifies neural ensembles that adjust to arousal level and stimulation frequency and evolve via dynamical rules **42**
J.1 Data and Pre-processing 42

J.2 Demonstration of Extended MILCCI With Non-Linear Transformation and Dynamics Prior Over Traces Evolution 42

J.3 Hyperparameter Sensitivity Analysis 43

K Ethics Statement and LLM Usage **46**

A. Additional Fitting Details:

A.1. Initialization:

We initialize the components and traces using dictionary learning (Mairal et al., 2009) with sparsity on the components, using Sklearn.decomposition (Pedregosa et al., 2011) DictionaryLearning. Within the model, sparsity is applied through PyLops (Ravasi & Vasconcelos, 2020) SPGL1's solver (Van Den Berg & Friedlander, 2009).

A.2. Details about state similarity graph calculation:

MILCCI allows disentangling of categorical, continuous, and non-continuous ordinal categories. It further supports allowing the components to adjust across trials with label change in a way that captures the degree of similarity between the corresponding labels for each category. For example, if we assume neuronal ensembles gradually present compositional shifts over the course of learning, this requires capturing the gradual / ordinal order of trials. Another example is when a certain category represents a continuous variable (e.g., x-position of a stimulus), where again we would like to capture label relationships (i.e., distance between labels).

Hence, MILCCI augments the model with a set of label-driven graphs that are pre-calculated before the beginning of the iterative optimization process and are reused across iterations for smoother cross-component regularization that maintains label similarity. For each category (k), we build the graph $\boldsymbol{\lambda}^{(\mathrm{k})} \in \mathbb{R}^{\#\mathrm{k} \times \#\mathrm{k}}$, where #k is the number of unique options observed under category (k) (e.g., if category "choice" can be correct / incorrect, then #k=2). This graph captures the degree of similarity between its possible values.

For categorical labels, we use a constant value for the graph (e.g., $\lambda^{(\mathrm{k})}_{i,i'} = 1 \;\; \forall i, i'$).

For ordinal labels: We use a Gaussian kernel $\lambda^{(\mathrm{k})}_{i,i'} = e^{\frac{\|\mathrm{k}_i - \mathrm{k}_{i'}\|_2^2}{2\sigma^2}} \;\; \forall i, i'$, where k_i and $\mathrm{k}_{i'}$ are the i-th and i'-th option of category (k) (e.g., task difficulty 1 vs. 5). Notably, MILCCI supports integration of diverse graph calculation distance metrics, so one can easily use a different distance metric (not Gaussian) if they assume similarities between labels are captured differently.

After the graph calculation, we recommend normalizing the graph by the per-row absolute sum of 1 to ensure that different labels are regularized to the same degree:

$$\lambda^{(\mathrm{k})}_{i,:} \leftarrow \frac{\lambda^{(\mathrm{k})}_{i,:}}{\|\lambda^{(\mathrm{k})}_{i,:}\|_1} \;\; \forall i, i'.$$

An i-th row of zeros in $\boldsymbol{\lambda}^{(\mathrm{k})}$ means that the i-th option of category (k) is not regularized to be consistent with the others. This can be used if there is some intention to create completely trial-varying components that vary flexibly between trials, which is another feature MILCCI offers.

A.3. About sparsity and consistency hyperparameters

Like most machine learning models, MILCCI includes hyperparameters that control model behavior, though notably fewer than complex models such as deep networks. Two key hyperparameters in MILCCI control component behavior: γ_1 (sparsity) and γ_2 (cross-label consistency).

γ_1 controls the ℓ_1 regularization on component memberships (Eq. 2). Higher γ_1 values produce sparser components with fewer non-zero entries, which is crucial for interpretability but potentially missing weaker relationships. Lower γ_1 values allow denser components that capture more subtle patterns but may include noise. γ_2 promotes similarity between component variants within the same category via ℓ_2 regularization on their distances (Eq. 2). Higher γ_2 values force variants to remain nearly identical, losing the ability to capture label-driven adjustments. Lower γ_2 values allow more flexibility but risk components diverging too much across labels.

We recommend selecting γ_1 by examining component sparsity and interpretability (e.g., inspecting the number of non-zero entries and their meaningfulness), and by testing information criteria (e.g., AIC, BIC) post-training. For γ_2, we recommend analyzing the distribution of pairwise distances between same-category component variants to ensure they remain similar yet allow meaningful adjustments. When domain knowledge is available (e.g., in neuroscience, we may have an estimate of

how many neurons form a group based on the amount of data we recorded), this can further guide hyperparameter selection.

B. Running Details

B.1. Data and Code Availability

All real-world data used in this paper are publicly available online, with sources cited in the corresponding sections. Code for the model implementation and synthetic data generation will be made available upon publication.

B.2. Versions

We trained the model using Python 3.10.4 (conda-forge) with matplotlib 3.8.2, scikit-learn 1.0.2, seaborn 0.11.2, numpy 1.23.5, pandas 1.5.0, PyLops 1.18.2, and SPGL1 0.0.2.

C. Elastic Net Prior of Components

In Section 3.1, we specified that the component matrices follow a Laplace distribution:

$$\boldsymbol{A}_{nj}^{(\mathrm{k})} \sim \mathrm{Laplace}(0, \frac{1}{\gamma_1}) = \frac{\gamma_1}{2} \exp\left(-\gamma_1 |\boldsymbol{A}_{nj}^{(\mathrm{k})}|\right)$$

When we later extended the model to multiple component variants for each category k with constrained ℓ_2 distances between them (i.e., $\|\mathcal{A}_{n:i}^{(\mathrm{k})} - \mathcal{A}_{n:i'}^{(\mathrm{k})}\|_2 < \epsilon$), we essentially employ a hierarchical Bayesian framework. The variant-specific components $\mathcal{A}_{n:i}^{(\mathrm{k})}$ thus follow an elastic net prior:

$$p(\{\mathcal{A}_{n:i}^{(\mathrm{k})}\}) \propto \exp\left(-\gamma_1 \sum_{n,j} |\mathcal{A}_{nji}^{(\mathrm{k})}| - \gamma_2 \sum_{i' \neq i} \lambda_{i',i}^{(\mathrm{k})} \|\mathcal{A}_{::i'}^{(\mathrm{k})} - \mathcal{A}_{::i}^{(\mathrm{k})}\|_2^2\right)$$

The first term corresponds to the ℓ_1 penalty inherited from the Laplace prior, promoting sparsity. The second term introduces an ℓ_2 penalty on variant differences, encouraging similarity within variant groups. This combination yields elastic net regularization, emerging naturally from the hierarchical structure where variants share statistical strength through their common base component.

D. Additional Information—Synthetic Experiment

We generated synthetic datasets with **80 channels**, **4 components** (2 categories, $\times$ 2 components adjusting per category), **500 time points per trial**, and **250 trials**. Each trial received one label per axis: category (a) (difficulty)'s labels were sampled from the set $\{\mathrm{I}, \mathrm{II}, \mathrm{III}, \mathrm{VI}, \mathrm{V}\}$ (5 levels) and category (b) (choice) labels from $\{\mathrm{I}, \mathrm{II}\}$ (2 levels), yielding up to **10 unique label combinations**. Component-to-neuron maps were initialized for the reference trial with values in $[0.5, 1.0]$, then updated across label variants according to a trial-similarity graph calculated based on trial labels and thresholded at the **60th percentile** to enforce sparsity on the component compositions.

Temporal activity for each label pair was drawn from a Gaussian-process prior with an RBF kernel scaled by a per-sample amplitude: the amplitude was drawn per sample in $\approx [0.2, 1.533]$, and the kernel length scale was drawn per sample in $[0.05, 0.2]$ in normalized time units (0–1), which corresponds roughly to **25–100 time points** given 500 samples per trial. A white-noise term of 1×10^{-8} was included in the kernel. For each label we drew one GP sample and then generated multiple similar trial traces by adding multivariate-normal perturbations with covariance scaled by σ^2, where $\sigma = 0.15$ ($\sigma^2 = 0.0225$), so trials that share a label exhibit correlated dynamics.

One component was designated as a random (trial-varying) component. Component activations were shifted to be nonnegative and rescaled so their 98th percentile matched the 98th percentile of the component maps. The observed data were produced

Algorithm 1 MILCCI Algorithm

Input: Observed trial data $\{\boldsymbol{Y}^{(m)}\}_{m=1}^{M}$, with associated multi-category labels $\{L^{(m)}\}_{m=1}^{M}$.
Pre-calculate: Label-similarity graph $\boldsymbol{\lambda}^{(\mathrm{k})}$ for each category (k) (App. A.2).
Initialize: Sparse components $\{\mathcal{A}^{(\mathrm{k})}\}_{\mathrm{k}\in\mathrm{Categories}}$ and traces $\{\boldsymbol{\Phi}^{(m)}\}_{m=1}^{M}$ (App. A.1)
repeat
  for each category (k) **do**
    for each label value k_i **do**
      Compute residuals for trials with label k_i
      Solve for $\mathcal{A}^{(\mathrm{k})}_{::i}$ with cross-label consistency and sparsity via LASSO ((2))
      Normalize each component to sum to 1 ($\mathcal{A}^{(\mathrm{k})}_{:ji} \leftarrow \frac{\mathcal{A}^{(\mathrm{k})}_{:ji}}{\|\mathcal{A}^{(\mathrm{k})}_{:ji}\|_1}$) to prevent scaling ambiguity with Φ
    end for
  end for
  for each trial m with label ℓ **do**
    Build the stacked component matrix $\boldsymbol{A}^{(\ell)}$ by selecting a variant from each $\mathcal{A}^{(\mathrm{k})}$
    Update traces $\boldsymbol{\Phi}^{(m)}$ to minimize data fidelity, smoothness and de-correlation ((3))
  end for
until converged

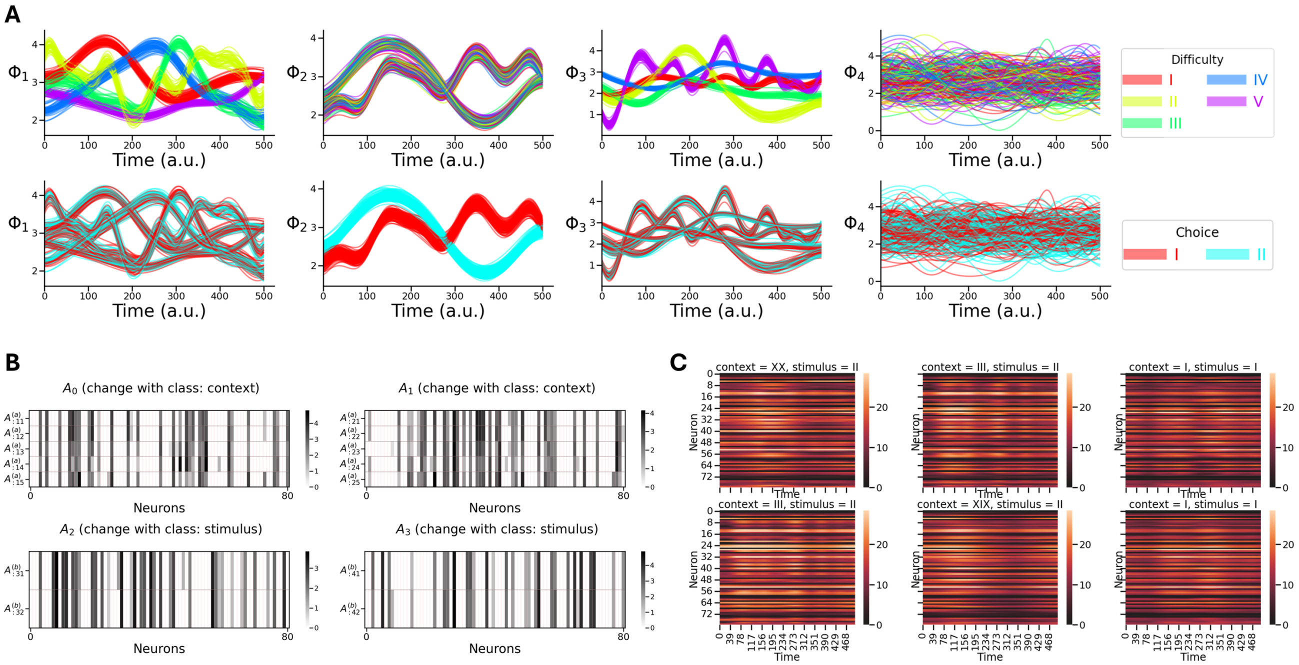

Figure 6. **Generated Synthetic Data. A:** Generated traces, colored by difficulty (top) or choice (bottom). **B:** Generated components. Each subplot shows one component and how it varies over the labels of each category (changes across rows). In other words, each subplot corresponds to $\mathcal{A}^{(\mathrm{k})}_{:j:}$ for some component j. **C:** Random example generated synthetic trials $\{\boldsymbol{Y}^{(m)}\}$.

by multiplying each trial's component-to-neuron map by that trial's temporal activations, yielding data of shape **(neurons** $\times$ **time** $\times$ **trials) = (80** $\times$ **500** $\times$ **250)**.

E. Additional Information—Voting Experiment

E.1. Voting Data Pre-Processing

Data were acquired from (Data & Lab, 2017a;b;c), which included vote information for presidential, senate, and house elections in 51 states, including Washington, DC. The datasets cover the years 1976 to 2020 for presidential and senate

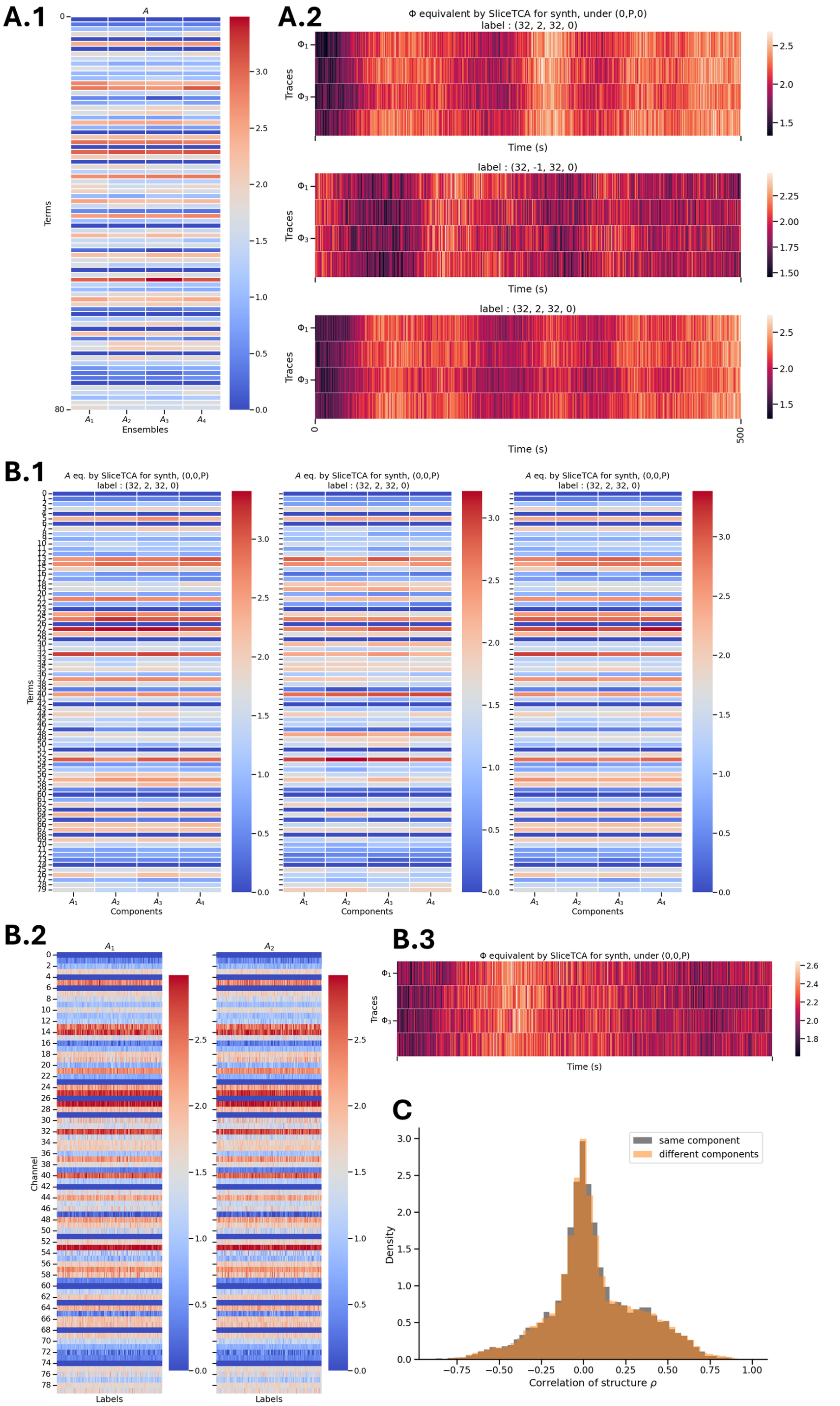

Figure 7. **Components and Traces Identified by SliceTCA on synthetic data. A** Results for configuration 1 (Sec. H.2), where **A.1** represents the components and **A.2** represents the corresponding traces from sliceTCA's $\boldsymbol{A}$ matrix. **B** Results for sliceTCA's configuration 2. **B.1** Identified components for 3 example trials; each subplot represents all components of one trial. **B.2** Shows how identified components vary over trials. **B.1 & B.2** together show that sliceTCA identifies components that are very close to each other, with cross-component variability similar to the variability of the same component over trials. This suggests that components are not necessarily matched over trials in terms of identity, as seen in panel **C** which shows the correlation distribution between same components and different components. **B.3** The temporal traces obtained by sliceTCA for configuration 2, shared across all trials.

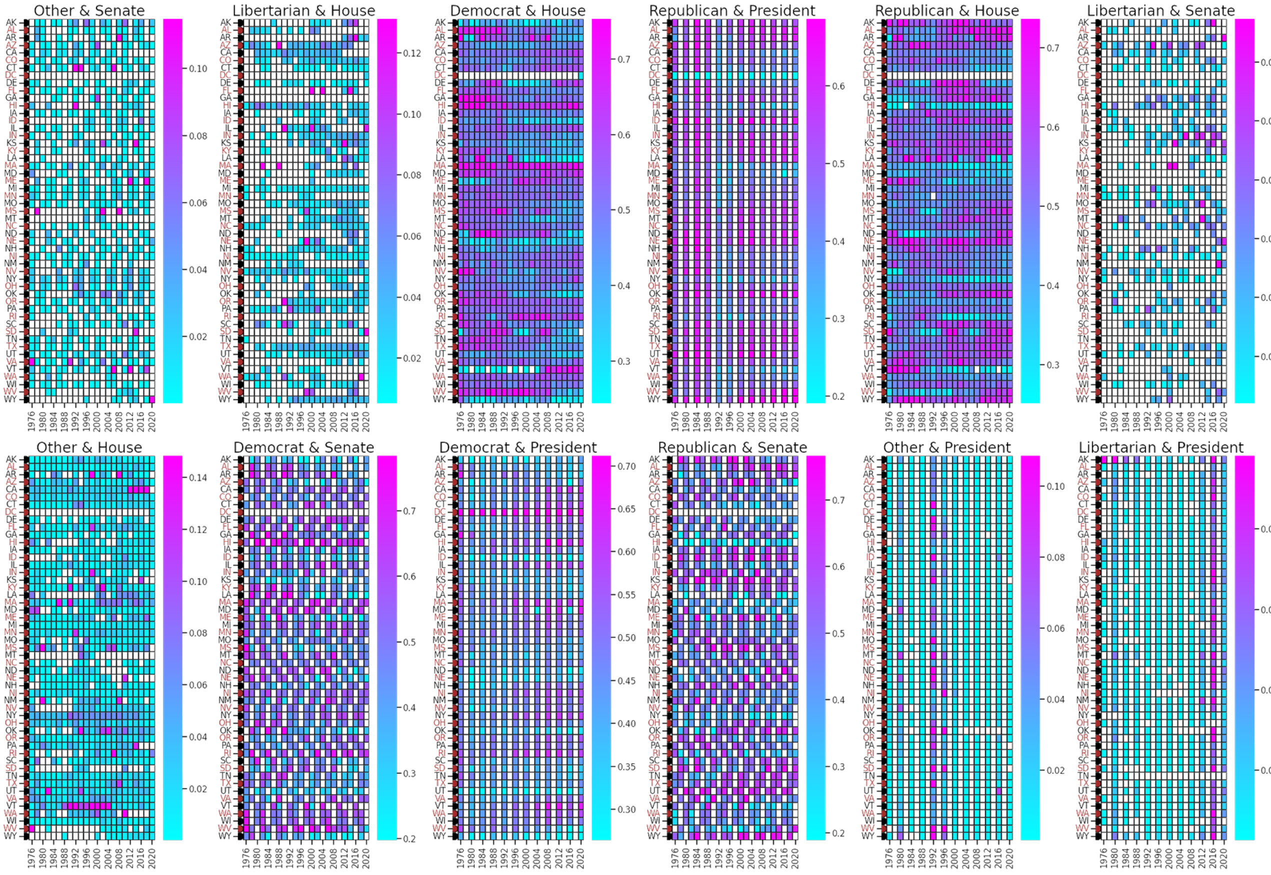

Figure 8. **Voting Data**: ($\{\boldsymbol{Y}^{(m)}\}_{m=1}^{M}$) Show Diverse Data Structures

elections, and 1976 to 2022 for house elections. For our analysis, we used the range 1976 to 2020 to ensure that all office types were included, resulting in 23 time points for house and senate, and 12 time points for presidential elections. For each year and each state, we took the total number of votes received by each party category (democrat, libertarian, republican, other) and divided each by the total number of votes cast in that state for that year's election. We excluded special elections. We designed the model to capture two following categories: 1) party (4 options), and 2) office (3 options). We ran MILCCI with $p^{(\mathrm{k})} = 4$ components for each category, that can structurally adjust per class, resulting in $p = 8$ total components. Due to the positive-only nature of the data, we applied a non-negativity constraint on both the unit-to-component memberships and the temporal traces.

E.2. Additional Findings—Voting Data

Some more interesting voting patterns, beyond these discussed in the main text, can be found in Figs. 15 and 11.

In component $\mathcal{A}^{(\text{party})}_{:4:}$, Washington DC appears as its own distinct cluster. This likely reflects its highly unique political profile as the nation's capital, overwhelmingly Democratic, with very high voter turnout, which differs significantly from other states. In $\mathcal{A}^{(\text{office})}_{:2:}$ (i.e., the 6-th component overall), which unlike previous components changes its composition depending on the office, West Virginia (WV) is included for House and Senate voting but excluded for the Presidency. This component reflects strong Democratic dominance in those legislative elections. The distinction for WV may align with its historical voting pattern of supporting Democrats more in local and state-level offices (House and Senate), while trending more Republican in presidential elections, reflecting a split in voter behavior based on the office. When exploring all traces colored by party (Fig. 15 top), Democrat vs. Republican is the dominant axis, consistently driving the most prominent distinctions over time. In some traces ($\boldsymbol{\Phi}_{:\mathcal{G}_1^{(\text{party})}}$, $\boldsymbol{\Phi}_{:\mathcal{G}_2^{(\text{party})}}$, $\boldsymbol{\Phi}_{:\mathcal{G}_3^{(\text{party})}}$), a noticeable divergence emerges around the year 2000, suggesting that the two parties began to separate more sharply around that period. This trend may correspond to the aftermath of the 2000 presidential election dispute (Bush vs Gore), which catalyzed a lasting partisan split, possibly further

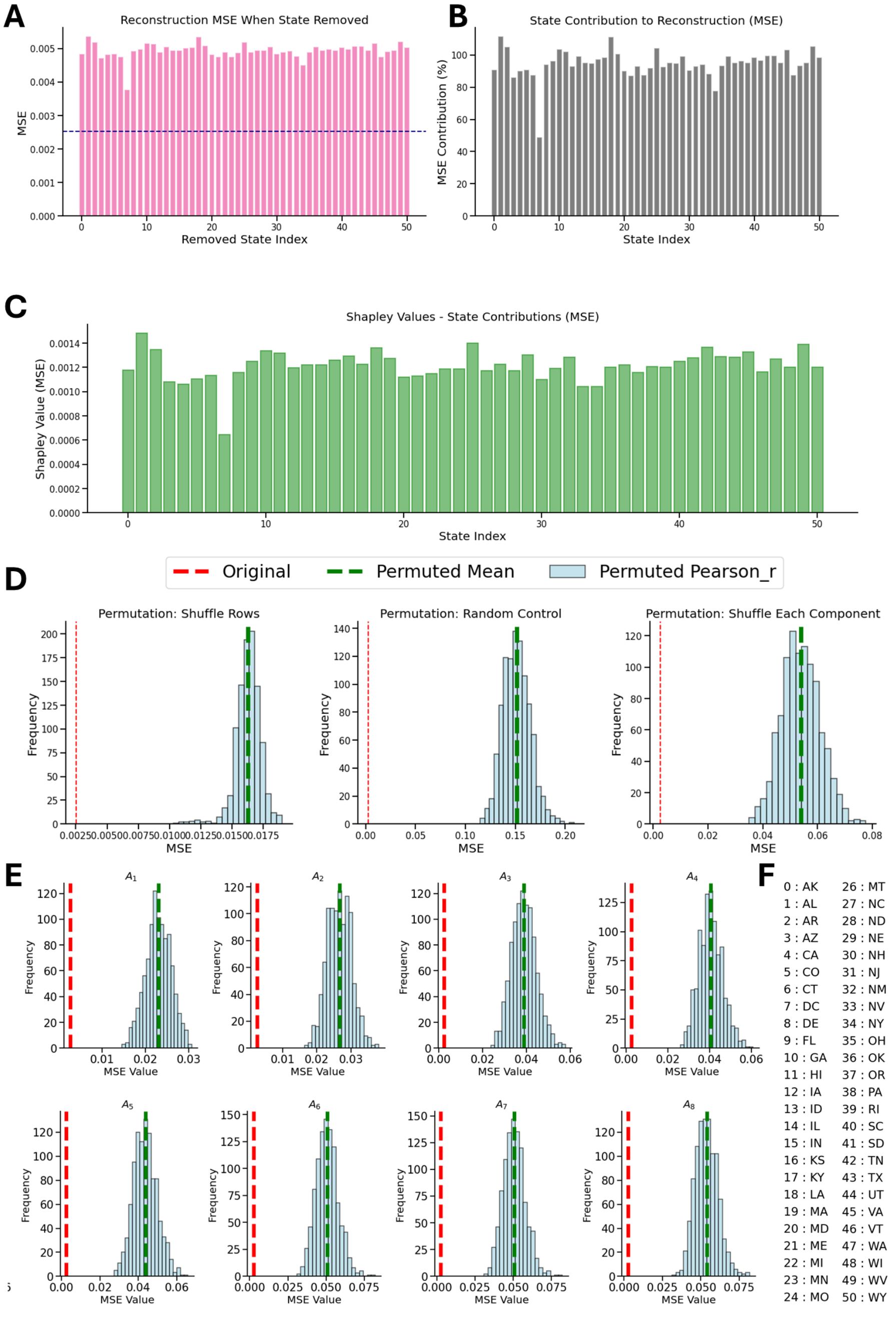

Figure 9. Post-hoc validation of MILCCI's discovered voting components (App. E.3). **A:** Leave-one-out analysis showing reconstruction MSE when each state is individually removed, with baseline MSE (dashed line) indicating model performance without omissions. **B:** Individual state contributions to reconstruction, how much each state's removal degrades performance ($100 * \frac{\text{MSE}_{\text{with omission}} - \text{MSE}}{\text{MSE}}$). **C:** Shapley values measuring each state's contribution to overall reconstruction. **D:** Permutation tests comparing original reconstruction error (red line) against three null hypotheses: shuffling state assignments between rows (left), replacing data with random noise (middle), and shuffling states within each component dimension (right). All tests show $p < 0.001$. **E:** Per-component permutation results demonstrating that discovered components are individually robust. **F:** State abbreviation key.

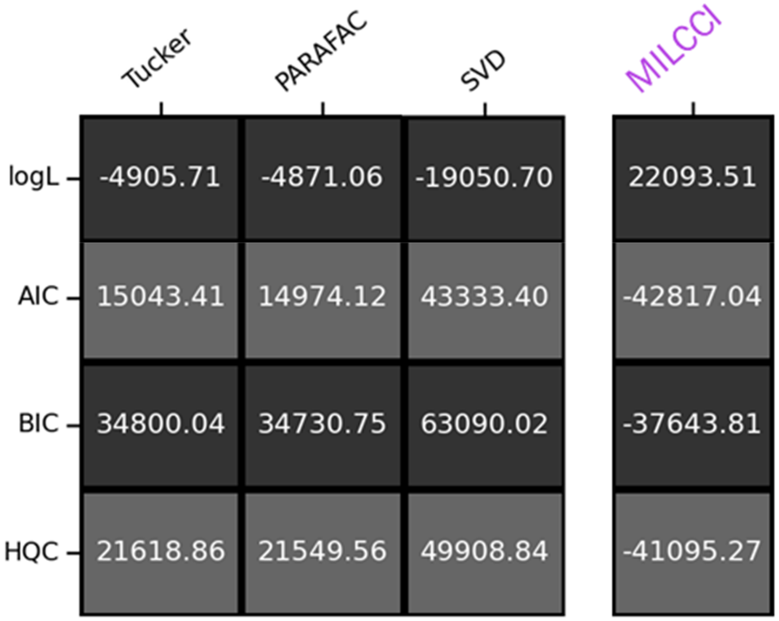

Figure 10. **Voting Experiment.** comparison to baselines in reconstruction and information criteria. Comparison to Tucker, PARAFAC, and SVD in terms of reconstruction and information criteria, including 1) log-likelihood of the observations given the identified components, and 2) information criteria that balance reconstruction and model complexity: AIC (Akaike Information Criterion), BIC (Bayesian Information Criterion), and HQC (Hannan-Quinn Criterion). Lower values indicate better performance.

intensified by the ideological polarization following 9/11. Other traces, such as $\mathbf{\Phi}_{:\mathcal{G}_2^{(office)}:}$ and $\mathbf{\Phi}_{:\mathcal{G}_4^{(office)}}$, show broader fluctuations over time in opposite directions, which may reflect deeper, long-standing historical or structural differences between the parties. Trace $\mathbf{\Phi}_{:\mathcal{G}_3^{(office)}}$ appears to capture short-term variation or noise, including year-to-year peaks in party voting behavior. In contrast, the projections based on the office (Fig. 15) exhibit far fewer separations. The traces overlap substantially and show wide confidence intervals, suggesting that electoral behavior is less differentiated by office type than by party affiliation.

E.3. Post-hoc Validation Analysis on Voting Data

To validate that MILCCI discovers genuine voting structure rather than spurious correlations, we perform comprehensive statistical analyses that examine individual state contributions and test against multiple null hypotheses (Fig. 9).

We remove each state from the component matrix by zeroing out that state's contribution and measure reconstruction degradation. For each of the N states, we calculate reconstruction MSE with the modified components where the target state is excluded. Fig. 9A shows the reconstruction MSE when each state is removed, with the baseline MSE (dashed line) that represents performance with all states included. Fig. 9B displays each state's contribution calculated as the percentage change in MSE relative to baseline performance.

We calculate each state's fair contribution with game-theoretic Shapley values, which consider all possible combinations of states. Due to computational constraints with N=52 states (which require 2^{52} calculations), we use approximation with 500 random combinations. For each combination, we include only combination members in the components matrix while we zero non-combination states, then calculate each state's marginal contribution as the difference in reconstruction quality when that state is added to the combination. Fig. 9C shows the Shapley values, which quantify each state's individual contribution to overall reconstruction quality.

We test three null hypotheses by comparison of original reconstruction performance against randomized versions:

Shuffle Rows (Fig. 9D, left):
We randomly reassign complete state voting profiles to different state positions. This tests whether the specific correspondence between geographic states and their voting behavioral patterns is meaningful.

Random Control (Fig. 9D, middle):
We replace the entire components matrix with random values drawn from a normal distribution with the same global mean

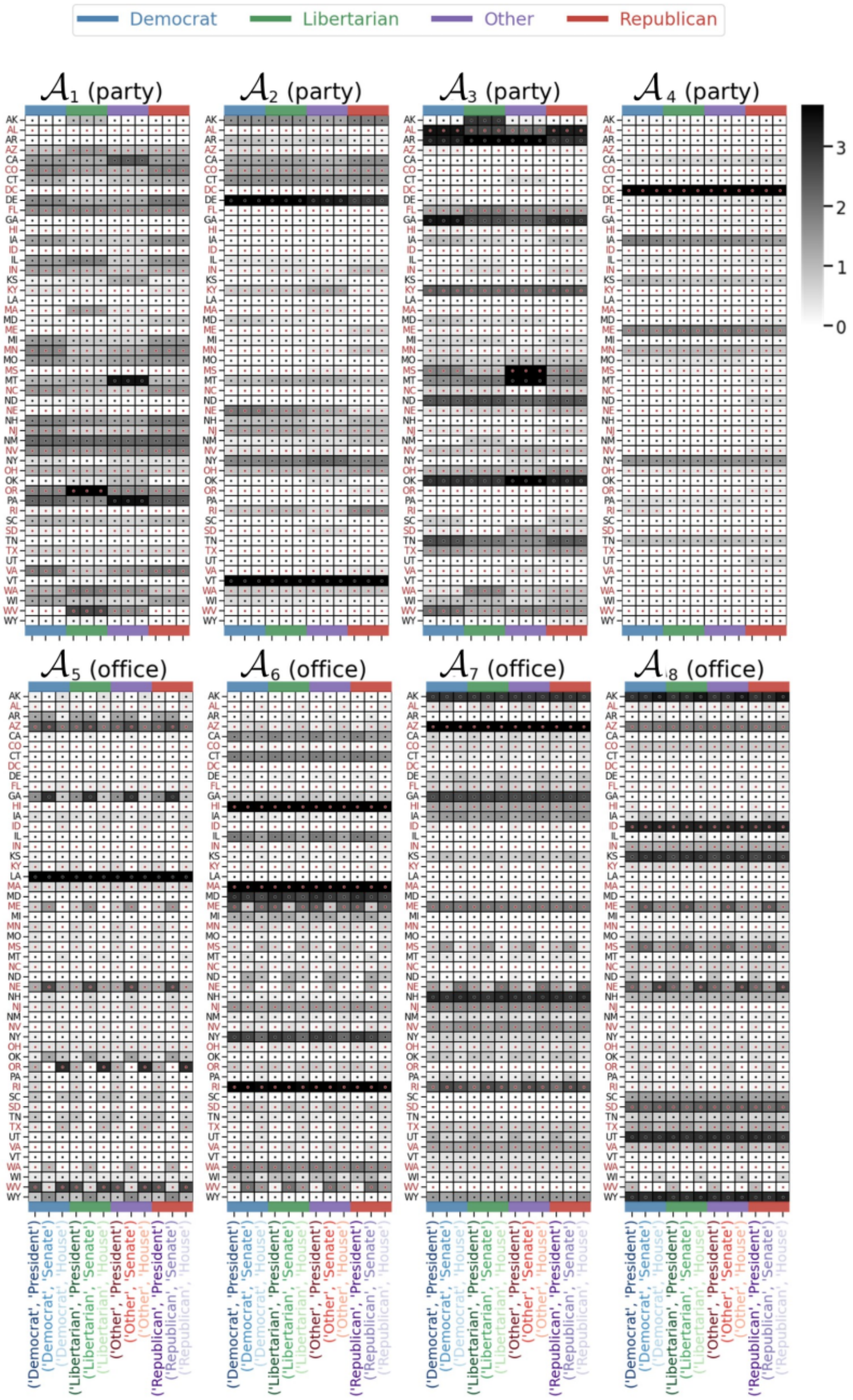

Figure 11. Identified Ensembles for Voting Experiment.

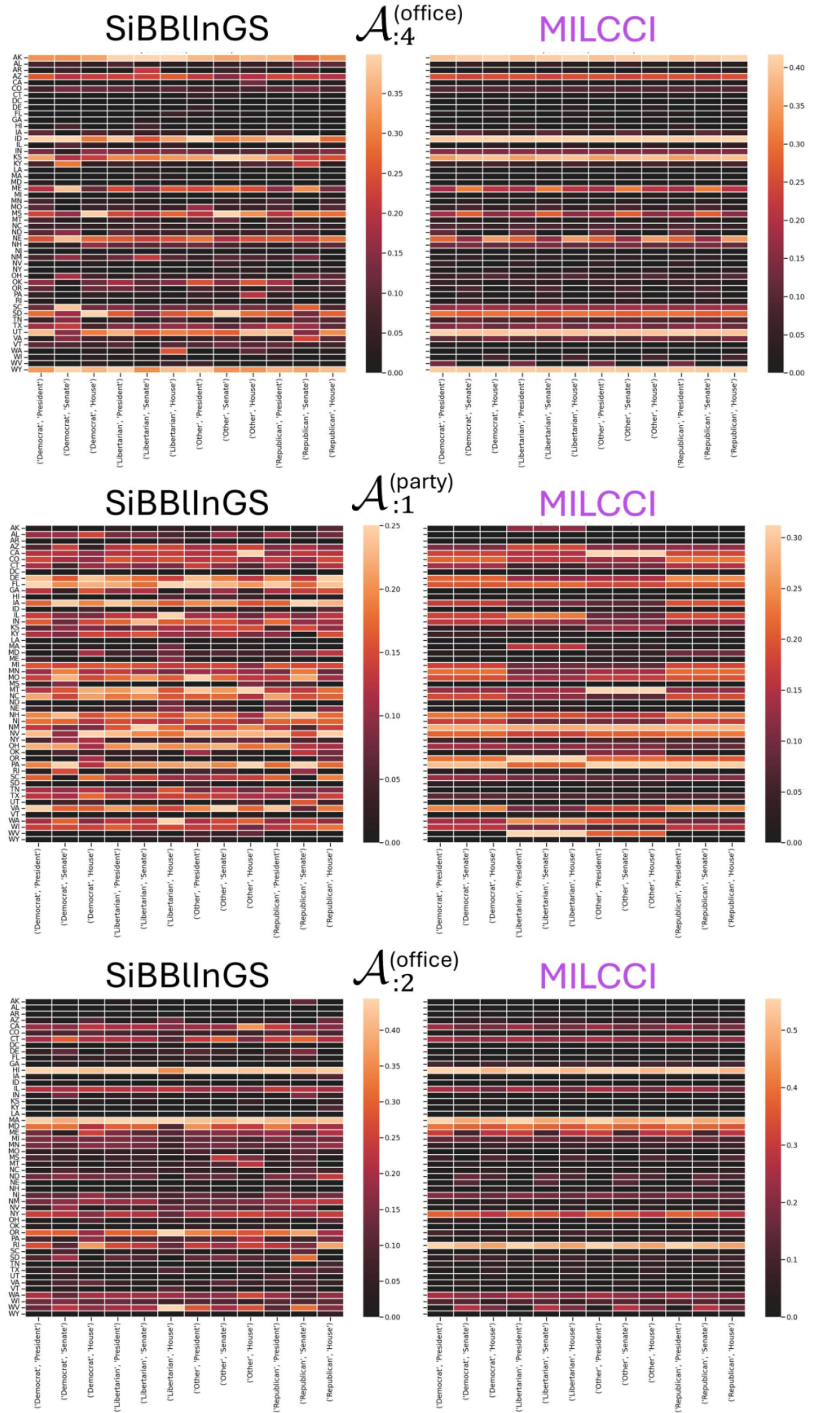

Figure 12. Comparison of components identified by MILCCI and SiBBlInGS for three example labels under the same parameters and seed. MILCCI components were duplicated to match the x-tick labels of SiBBlInGS. SiBBlInGS shows uninterpretable changes across every label, even when parts are shared, whereas MILCCI disentangles them.

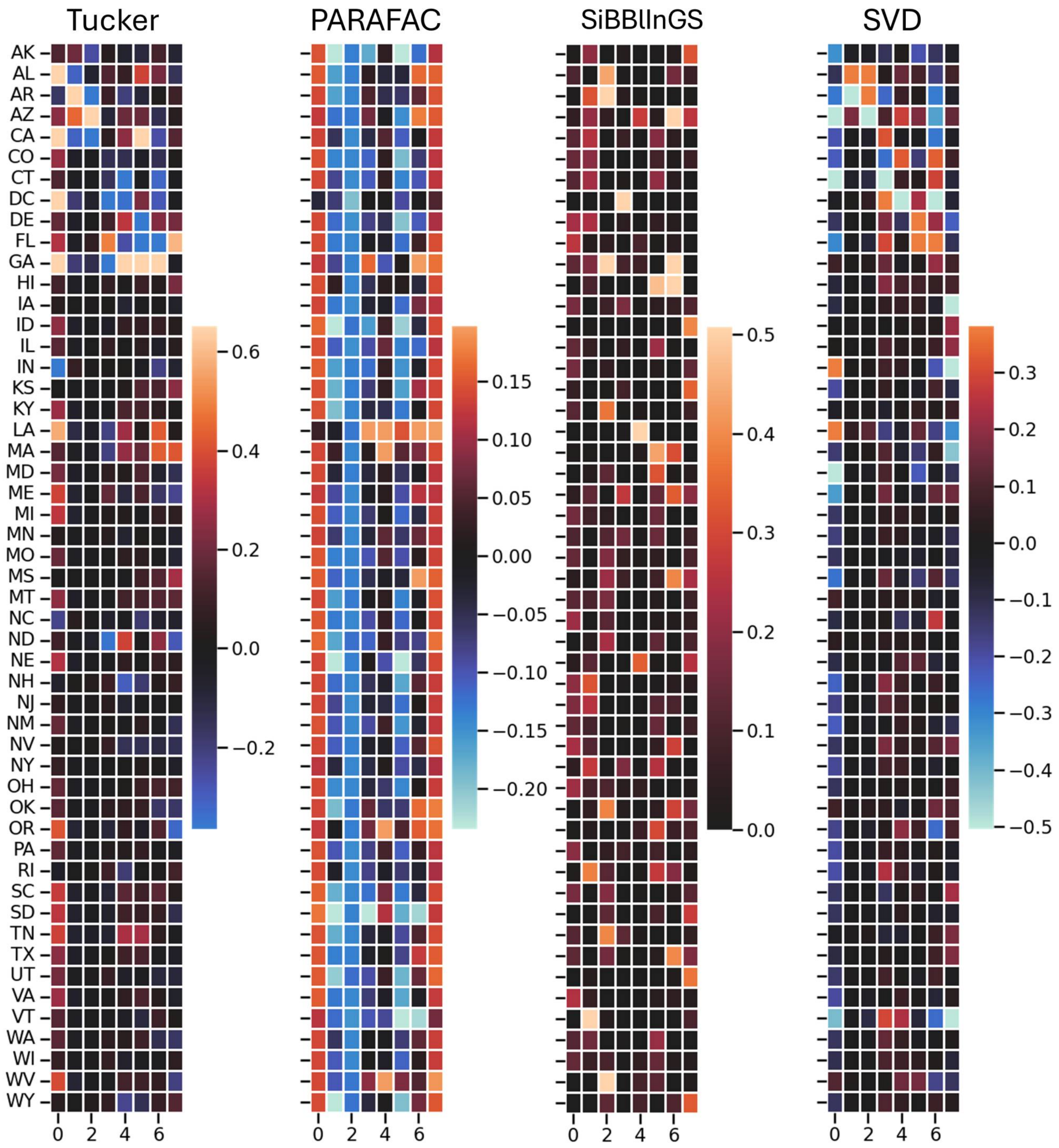

Figure 13. **Voting Experiment Baseline Comparison**. Components identified by the following baselines: 1) Tucker Decomposition (HOSVD), 2) PARAFAC, 3) SiBBlInGS (for a single random label entry: (Democrat, President)), 4) SVD (on all concatenated trials).

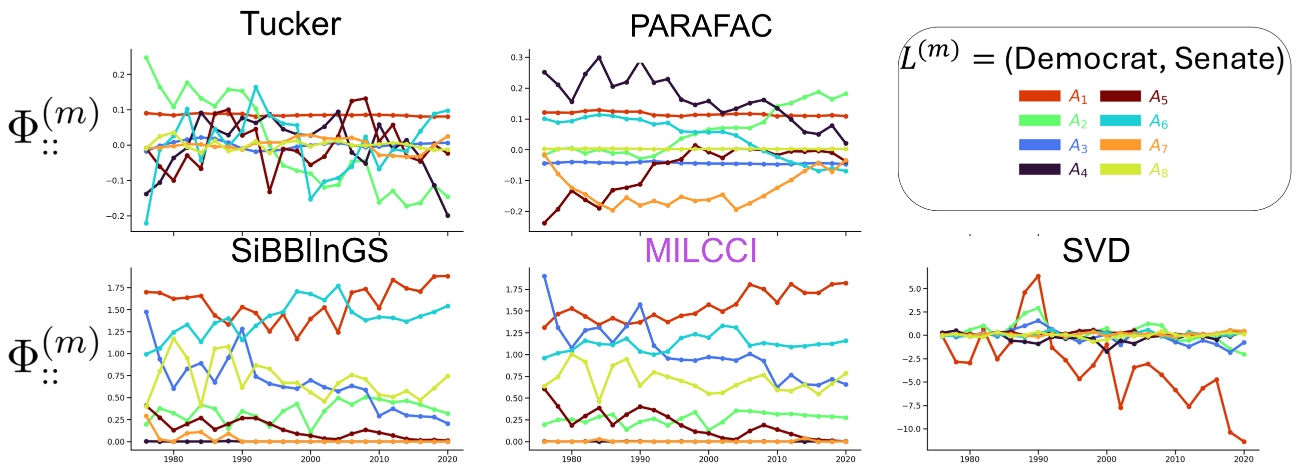

Figure 14. **Voting Experiment**. Traces Identified by MILCCI compared to the other baselines for example random trial.

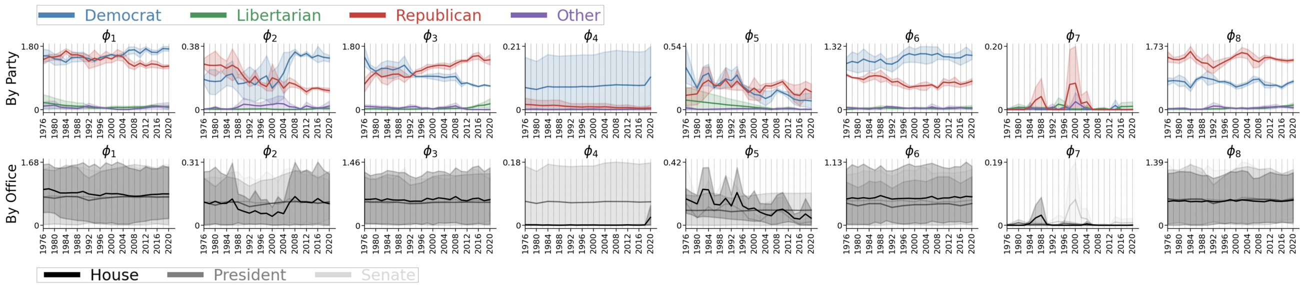

Figure 15. **Voting Traces.** Top: Colored by Party. Bottom: Colored by Office.

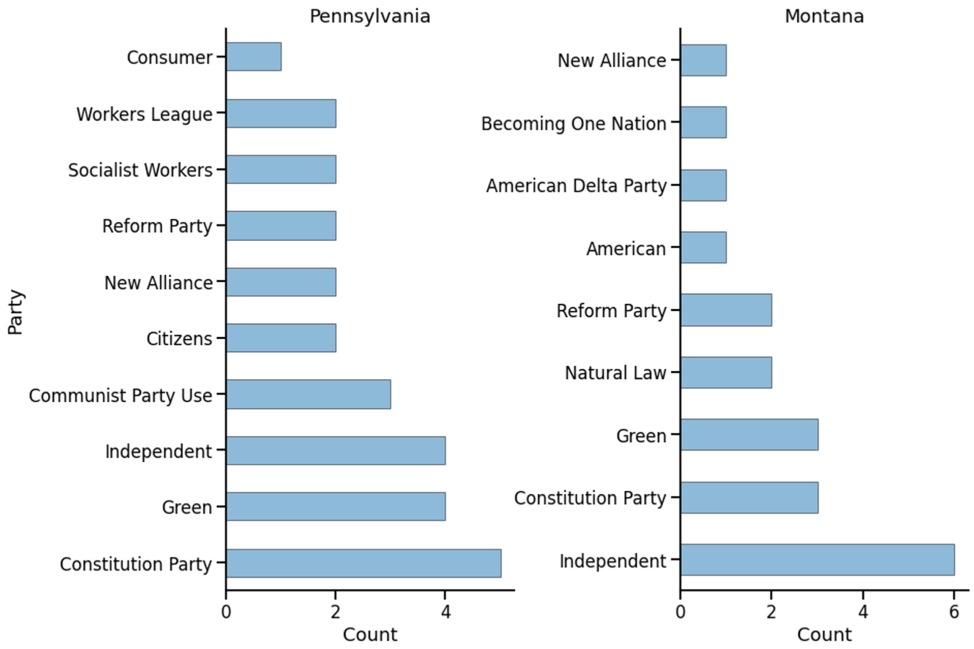

Figure 16. Top “Other” parties instance counts for Montana and Pennsylvania

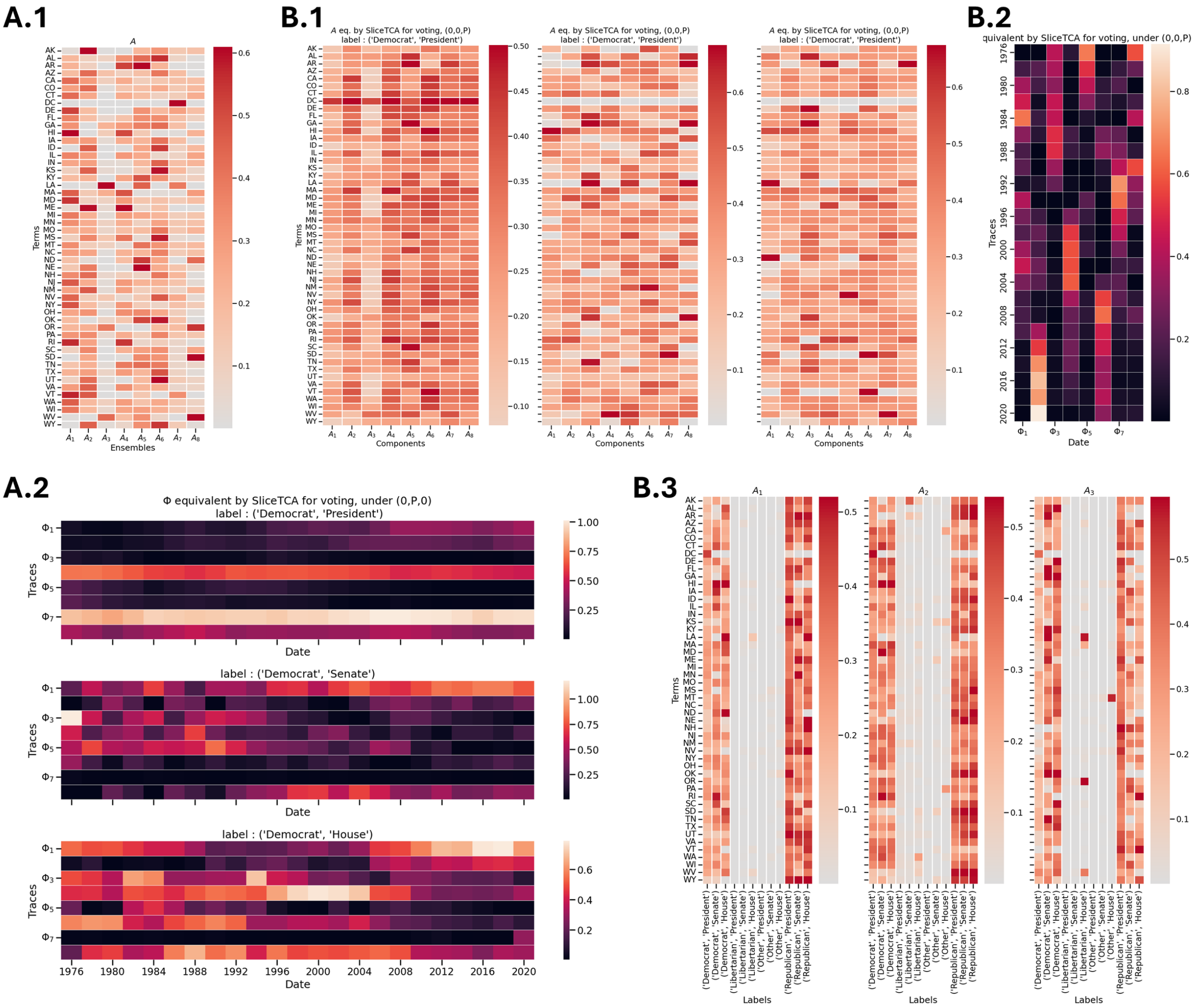

Figure 17. **Voting experiment: SliceTCA comparison. A.1** Components from the fixed component case (i.e., from u). **A.2** Traces from the fixed component case (i.e., from A). **B.1** Components from the varying component case (i.e., B). Each subplot represents a trial and columns show its different components. **B.2** Components from the varying component case (i.e., v). **B.3** Components from the varying component case. Each subplot represents one component and columns represent trials. We can observe that there is no consistency within the same component (B.3) across trials compared to across components.

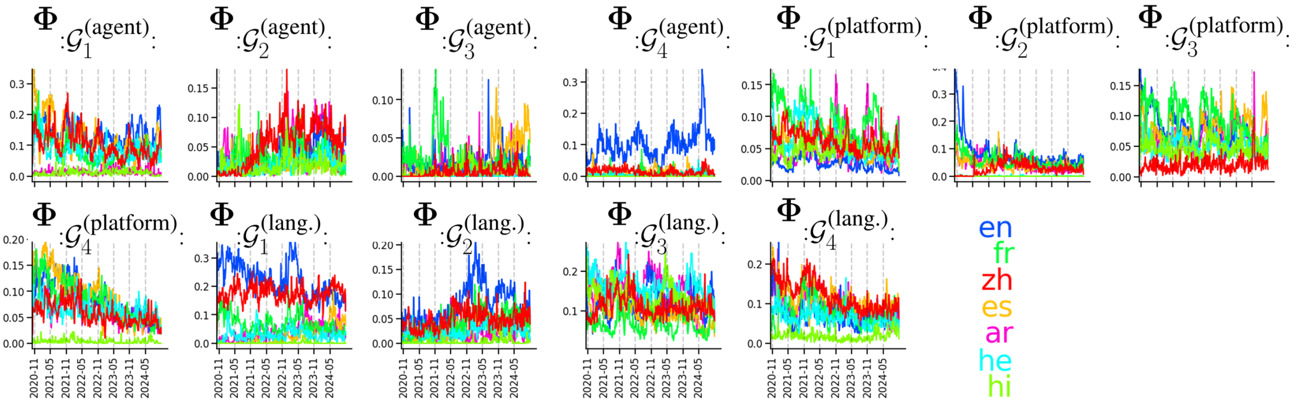

Figure 18. **Wikipedia Pageview Experiment**. Traces colored by Language (Arabic, English, Spanish, French, Hebrew, Hindi, Chinese) Across All Ensembles.

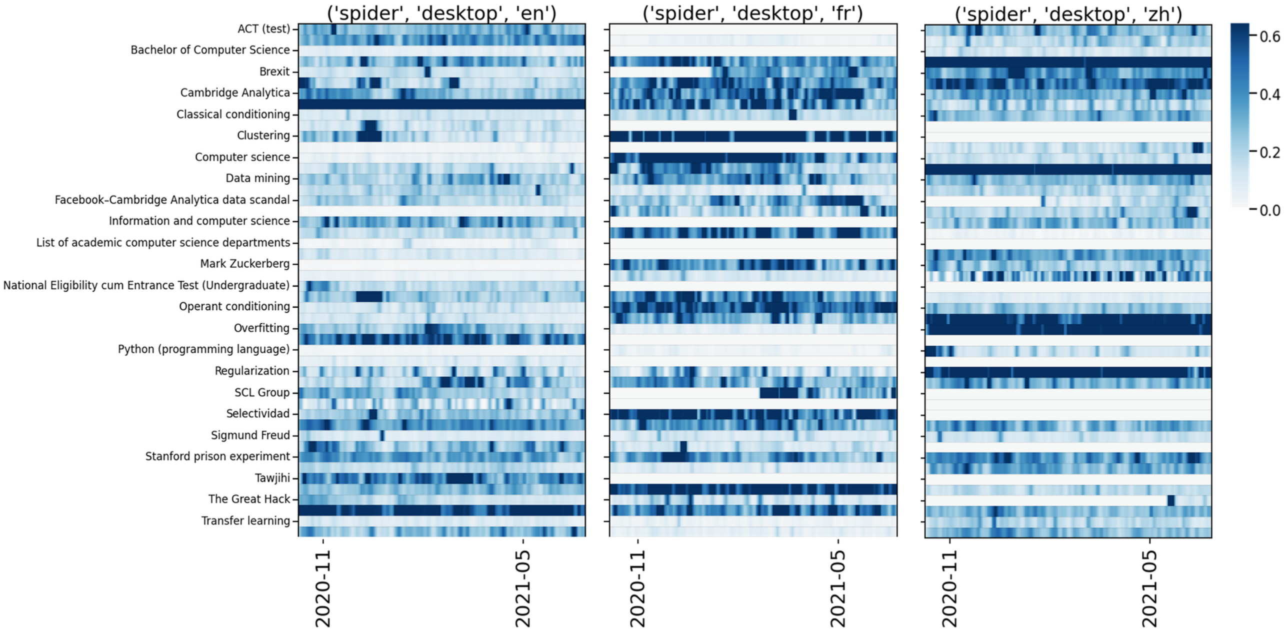

Figure 19. Wikipedia Pageview Data, example Trials

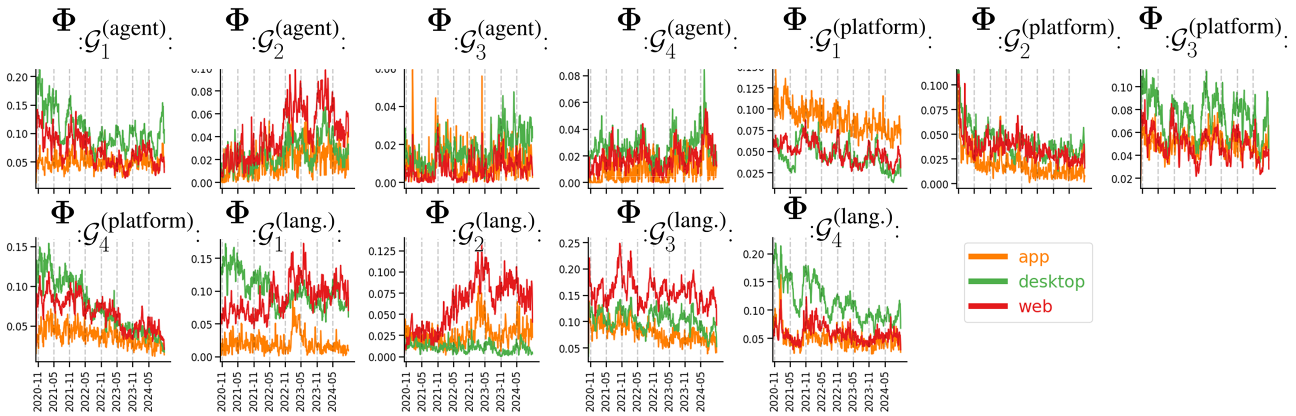

Figure 20. **Wikipedia Pageview Experiment**. Traces colored by Platform (desktop, mobile web, mobile app) Across All Ensembles

Abbreviation	State	Abbreviation	State
AL	ALABAMA	AK	ALASKA
AZ	ARIZONA	AR	ARKANSAS
CA	CALIFORNIA	CO	COLORADO
CT	CONNECTICUT	DE	DELAWARE
DC	DISTRICT OF COLUMBIA	FL	FLORIDA
GA	GEORGIA	HI	HAWAII
ID	IDAHO	IL	ILLINOIS
IN	INDIANA	IA	IOWA
KS	KANSAS	KY	KENTUCKY
LA	LOUISIANA	ME	MAINE
MD	MARYLAND	MA	MASSACHUSETTS
MI	MICHIGAN	MN	MINNESOTA
MS	MISSISSIPPI	MO	MISSOURI
MT	MONTANA	NE	NEBRASKA
NV	NEVADA	NH	NEW HAMPSHIRE
NJ	NEW JERSEY	NM	NEW MEXICO
NY	NEW YORK	NC	NORTH CAROLINA
ND	NORTH DAKOTA	OH	OHIO
OK	OKLAHOMA	OR	OREGON
PA	PENNSYLVANIA	RI	RHODE ISLAND
SC	SOUTH CAROLINA	SD	SOUTH DAKOTA
TN	TENNESSEE	TX	TEXAS
UT	UTAH	VT	VERMONT
VA	VIRGINIA	WA	WASHINGTON
WV	WEST VIRGINIA	WI	WISCONSIN
WY	WYOMING		

Table 2. List of US States and Their Abbreviations

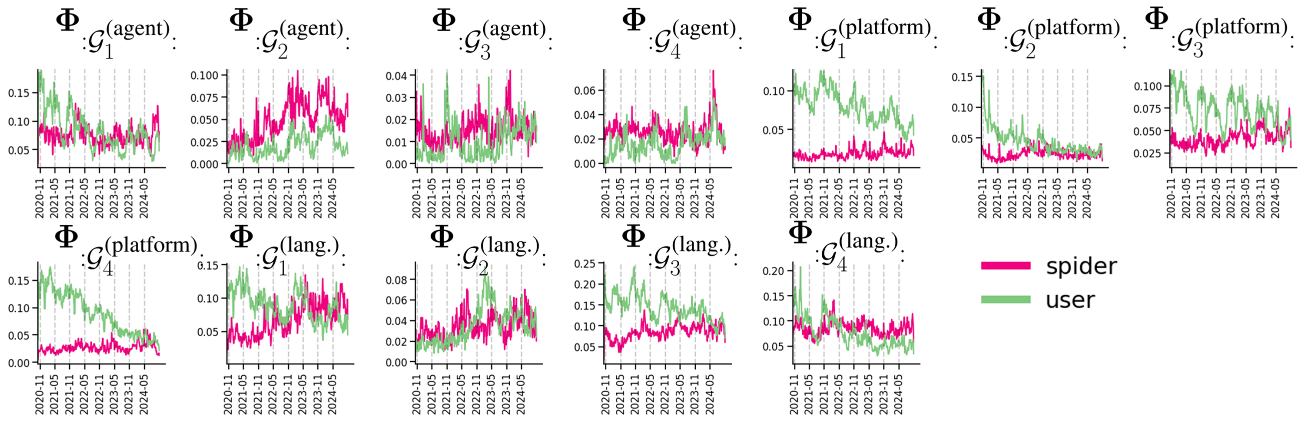

Figure 21. **Wikipedia Pageview Experiment**. Traces colored by agent (Spider vs. User) Across All Ensembles

and standard deviation as the original data. This tests against pure statistical noise baseline.

Shuffle Each Component (Fig. 9D, right):
For each component dimension, we randomly permute the assignment of states within that component while we preserve component-wise statistics. This tests whether the specific coordination between components within each state matters.

Each permutation test runs 1000 iterations, with p-values calculated as the fraction of permuted reconstructions that achieve equal or better performance than the original.

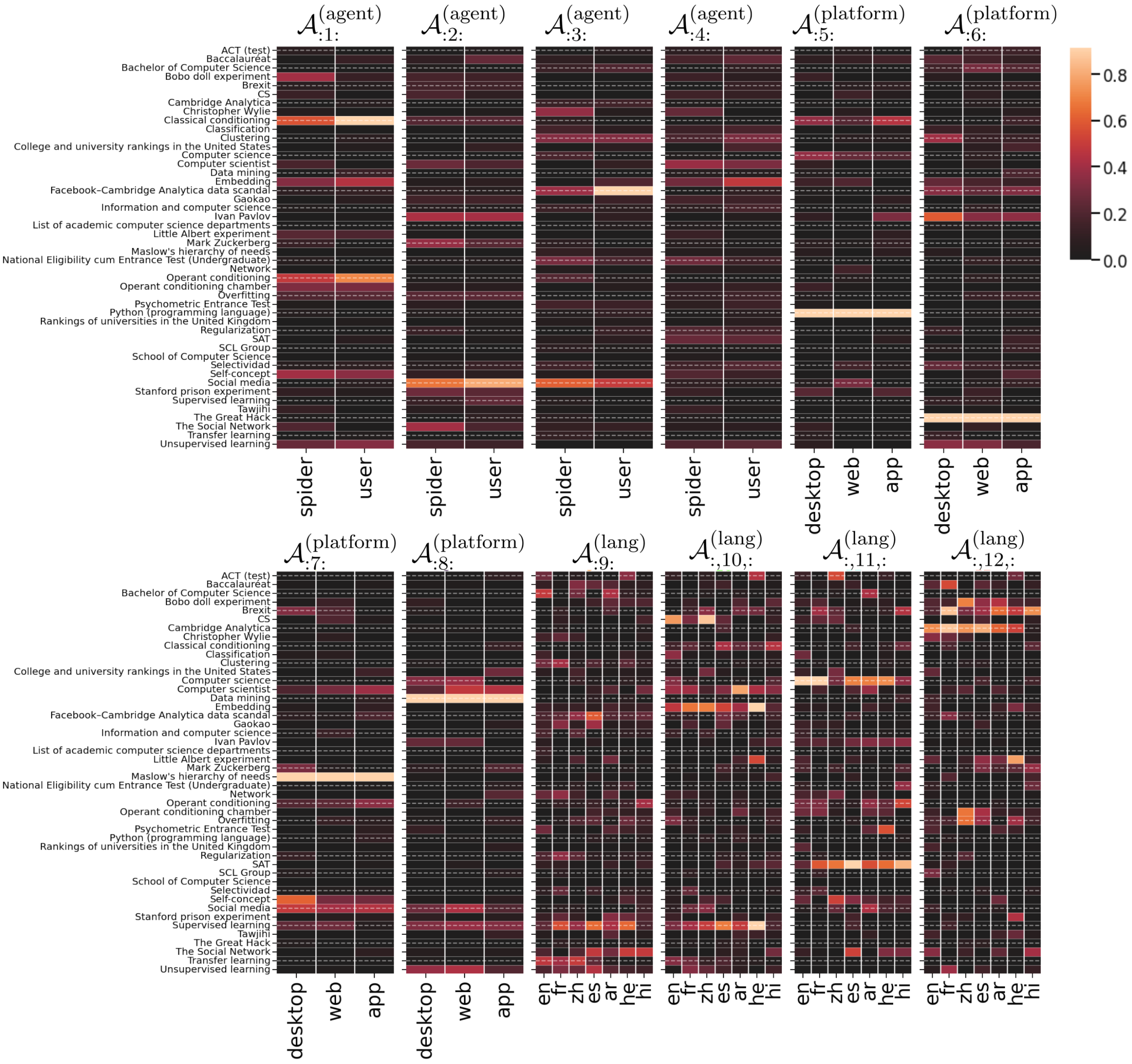

Figure 22. Components identified for Wikipedia Pageview Experiment.

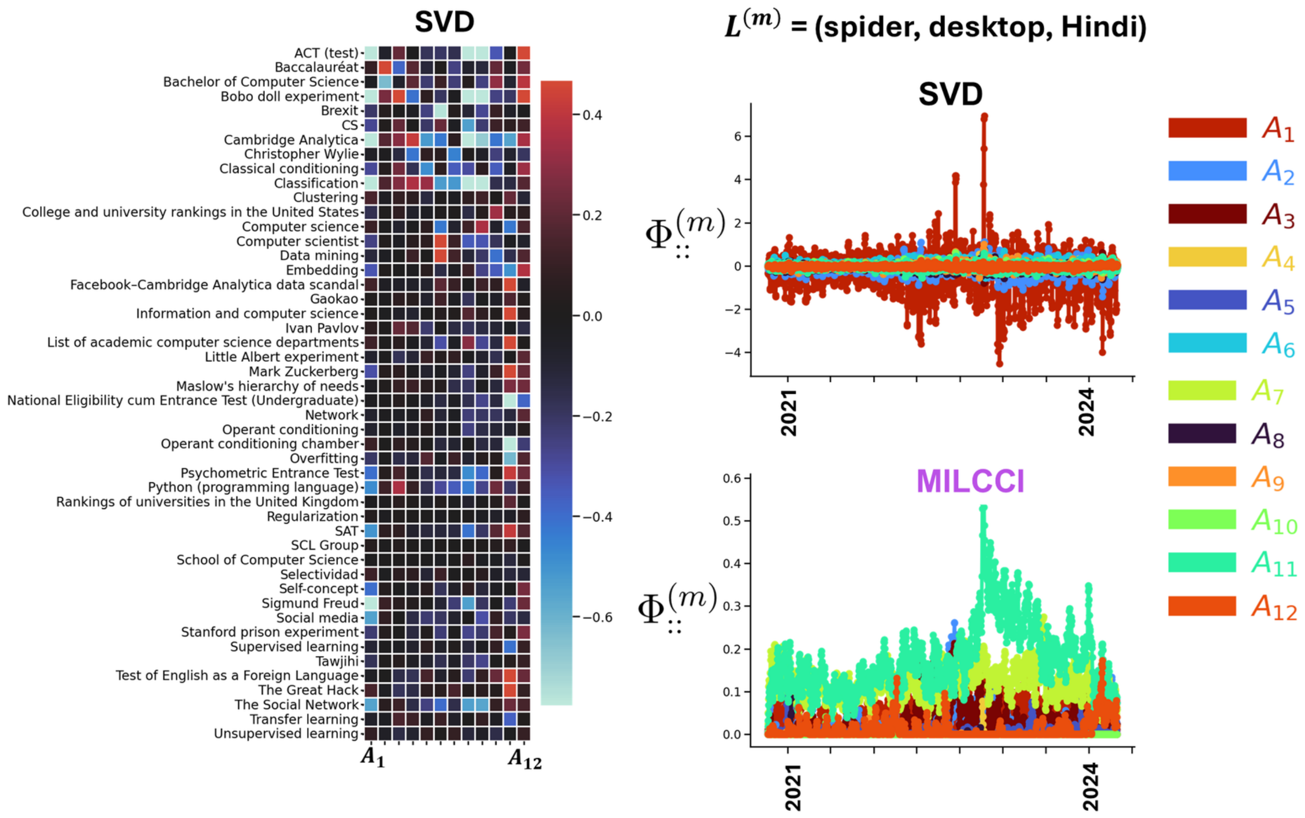

Figure 23. **Wikipedia Experiment Compared to SVD**. Components identified by SVD (compositions on the left, example trial traces on the right).

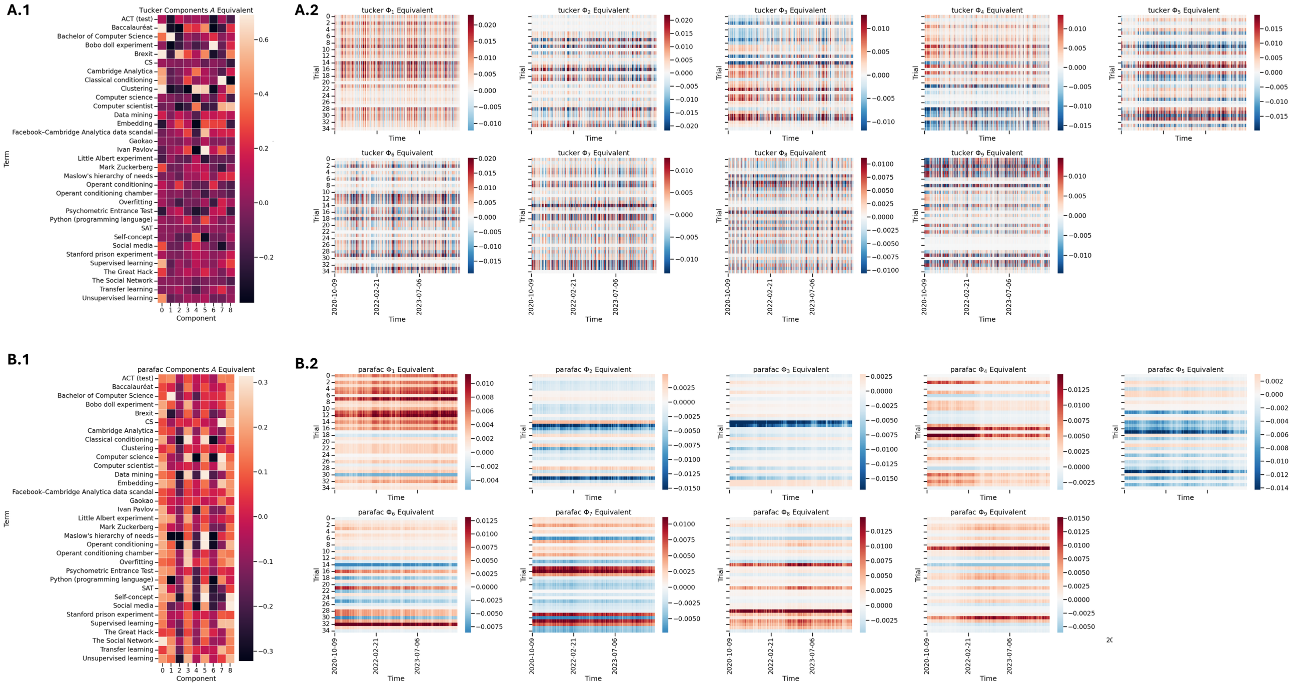

Figure 24. Components Identified by Tucker (**A.1**, **A,2**) and PARAFAC (**2.1**, **B.2**) for the Wikipedia Experiment, see App. H

Table 3. Parties Detailed vs. Simplified Versions. 'Other' includes only parties with at least 10 instances over all years & states.

Democrat	Republican	Other	Libertarian
Democrat; Democratic-Farmer-Labor; Democratic-Nonpartisan League; Democratic-Npl; Democrat (Not Identified On Ballot)	Republican	Prohibition; Independent; American Independent; U.S. Labor; Socialist Workers; American; Conservative; Socialist Labor; Independent American; Constitution; Socialist; Liberty Union; Statesman; Citizens; New Alliance; Workers World; Workers League; Independence; Populist; Nominated By Petition; Grassroots; No Party Affiliation; Green; Natural Law; Unaffiliated; Other; Working Families; Alliance; Non-Affiliated; Constitution Party; American Independent Party; Communist Party Use; Peace & Freedom; Taxpayers Party; Reform Party; U.S. Taxpayers Party; Socialism And Liberation Party; American Delta Party; American Solidarity Party; Party For Socialism And Liberation; Becoming One Nation	Libertarian

Component-specific permutation results (Fig. 9E) demonstrate that discovered patterns are robust across individual voting components. We store intermediate reconstruction results for each component, which allows examination of component-specific robustness to randomization. Fig. 9F provides the state abbreviation key for reference.

All statistical tests yield $p < 0.001$, which provides evidence that MILCCI's discovered voting patterns represent genuine structure.

F. Additional Information–Wikipedia Experiment

F.1. Wikipedia Pageview Data Pre-Processing

We extracted daily Wikipedia Pageview data from October 9, 2020, to October 29, 2024 ($T = 1482$ time points) for 48 diverse pages ("terms") related to college, computer science, machine learning, and psychology majors ((Meta, 2022)). Notably, we intentionally chose topics with both corollaries and co-variates. For each term, we collected data separately for three access platforms: (1) desktop, (2) mobile web, and (3) mobile app. We also distinguished the agent accessing the data: 1) a user or 2) a spider (for spider data was extracted only for web and desktop platform due to extreme sparsity of app + spider combination).

We focused on seven languages representing diverse world regions: English (en), Chinese (zh), Spanish (es), Hindi (hi), Arabic (ar), French (fr), and Hebrew (he). To ensure comparability, data for each language were range-normalized across all terms and time points using the 99th percentile to reduce outlier influence: $\boldsymbol{Y}_{:,:,l} \leftarrow (\boldsymbol{Y}_{:,:,l} - \min(\boldsymbol{Y}_{:,:,l}))/\text{perc}(\boldsymbol{Y}_{:,:,l}, 99)$, where $Y_{:,:,l}$ is the full dataset for language l.

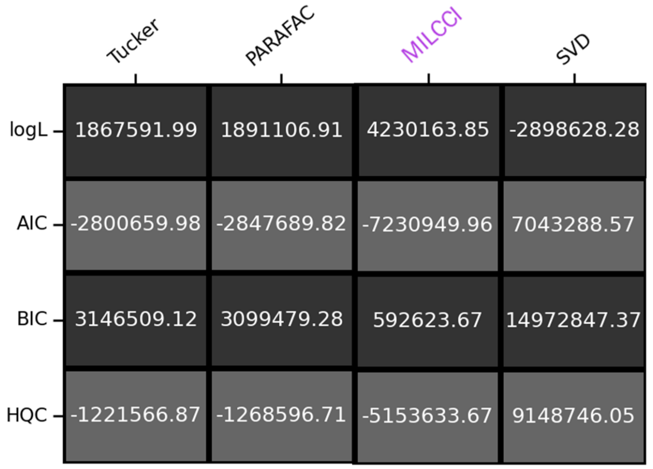

	Tucker	PARAFAC	MILCCI	SVD
logL	1867591.99	1891106.91	4230163.85	-2898628.28
AIC	-2800659.98	-2847689.82	-7230949.96	7043288.57
BIC	3146509.12	3099479.28	592623.67	14972847.37
HQC	-1221566.87	-1268596.71	-5153633.67	9148746.05

Figure 25. Information Criteria for Wikipedia Experiment, MILCCI vs. baselines. Notably, PARAFAC and Tucker did not converge of 12 components due to SVD instability. Notably, PARAFAC and Tucker encountered instability issues, and hence the results presented for Tucker and PARAFAC here are for rank 9 and added noise with $\sigma = 0.1$ (Tucker) and $\sigma = 0.2$ (PARAFAC).

Each term was then normalized across all languages and time points using the same 99th percentile procedure: $\boldsymbol{Y}_{k,:,:} \leftarrow (\boldsymbol{Y}_{k,:,:} - \min(\boldsymbol{Y}_{k,:,:}))/\text{perc}(\boldsymbol{Y}_{k,:,:}, 99)$, where $\boldsymbol{Y}_{k,:,:}$ denotes the full time-course of term k across languages. This results in overall three categories candidate for compositional adjustments in the data: (1) agent (user or spider), (2) platform (desktop / mobile web / mobile app), and (3) language (one of the seven listed).

F.2. Clarification on Findings—Wikipedia Data

Lists of terms of components mentioned in main text (Sec. 4):

- $\mathcal{A}^{\textbf{(agent)}}_{:1:}$ **(Psychology)**: Classical conditioning; Bobo doll experiment; Operant conditioning; Self-concept; Little Albert experiment; Unsupervised learning; Embedding;
- $\mathcal{A}^{\textbf{(agent)}}_{:2:}$**: (Social Media)**: The Social Network (movie); Social media; Ivan Pavlov;Mark Zuckerberg.
- $\mathcal{A}^{\textbf{(platform)}}_{:4:}$**: (Computer Science)**: Data mining; Computer science; Supervised learning; Unsupervised learning; Computer scientist; Social media;

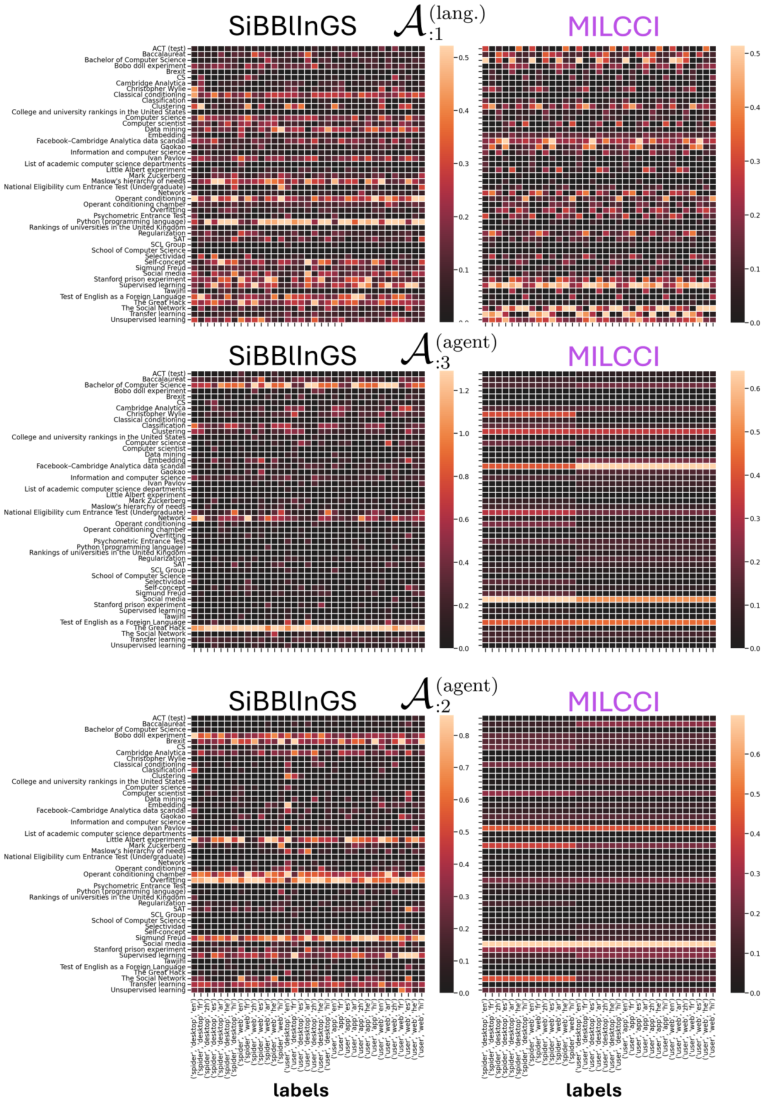

Figure 26. **Wikipedia Components, MILCCI vs. SiBBlInGS**. SiBBlInGS components display compositional changes scattered across labels, rather than the category-specific adjustments captured by MILCCI. *Note:* MILCCI components are shown here with duplicate columns to align with SiBBlInGS components for visualization.

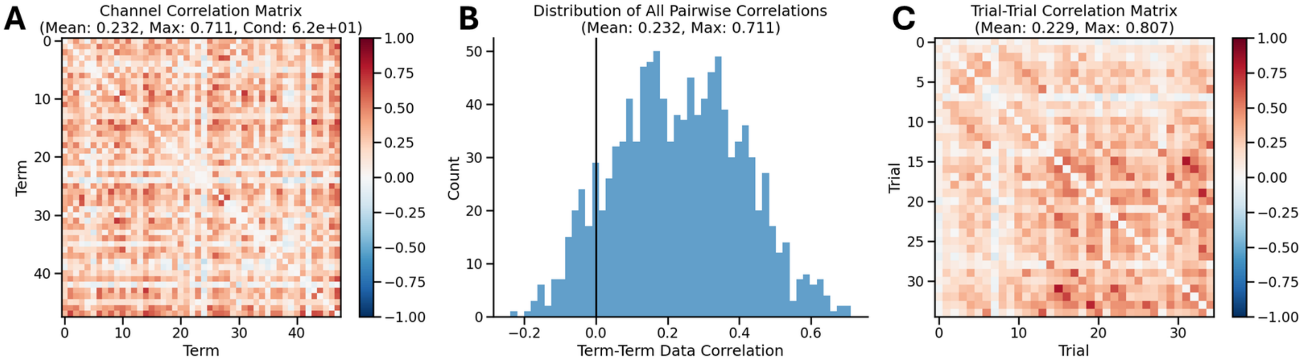

Figure 27. Correlation analysis of Wikipedia dataset reveals moderate correlations that do not explain baseline convergence failures. (A) Channel (term-term) correlation matrix shows moderate correlations with maximum of 0.711 and good condition number (6.2e+01). (B) Distribution of all pairwise correlations demonstrates that most correlations are moderate, with no high correlations (>0.8). (C) Trial-trial correlation matrix shows similar moderate correlation patterns (max 0.807). The data has full rank (48/48) and reasonable correlation structure, indicating that baseline convergence failures (Tucker, PARAFAC) stem from algorithmic limitations rather than problematic data characteristics.

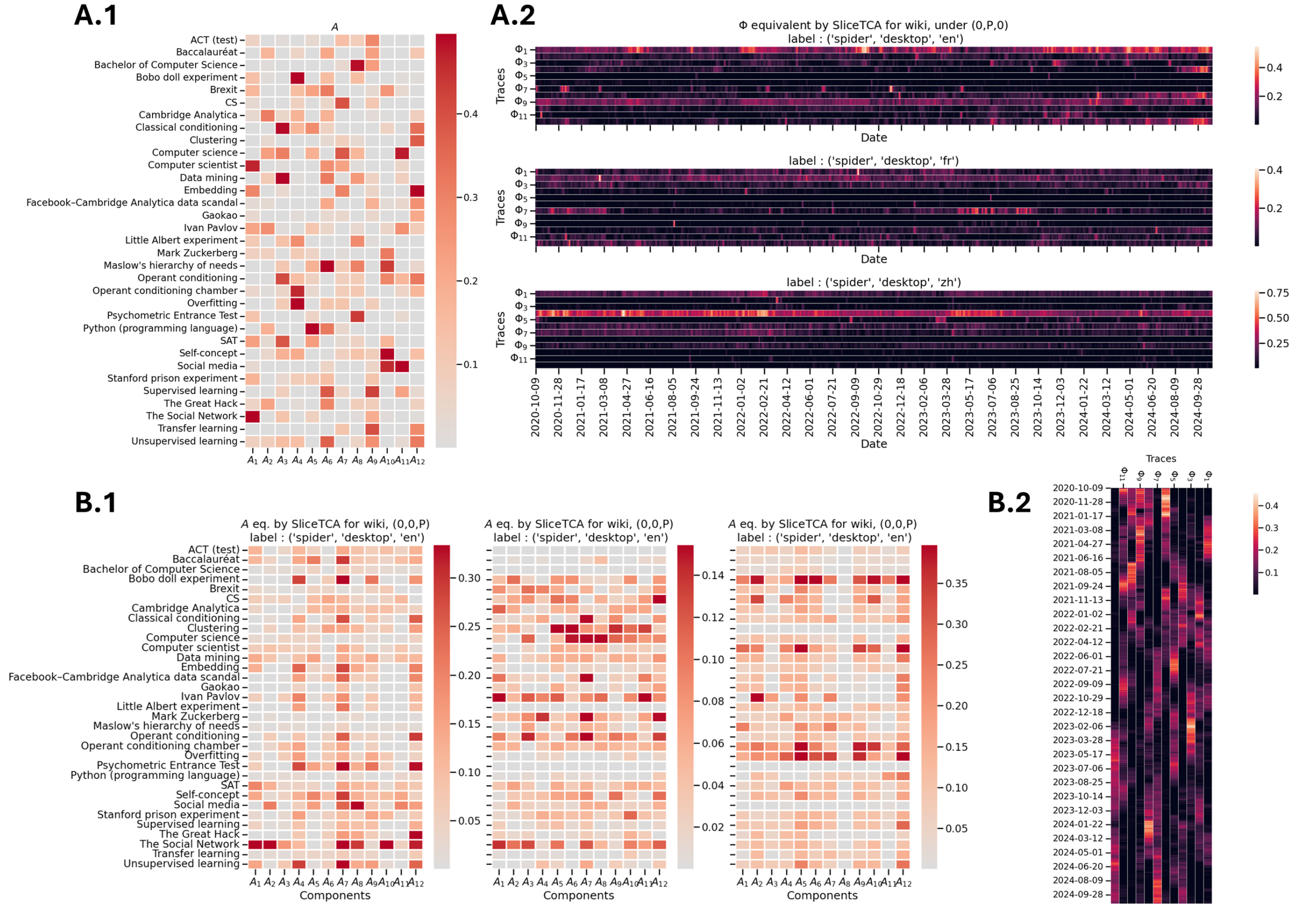

Figure 28. **Wikipedia experiment: SliceTCA comparison.** Components equivalent to MILCCI's were extracted from SliceTCA's configuration 1 (see Sec. H.2), i.e., components extracted from sliceTCA's $\boldsymbol{u}$ vector (**A.1**) and temporal traces from sliceTCA's $\boldsymbol{A}$ matrix (**A.2**). For the second configuration (**B** panels), components (MILCCI's $\mathcal{A}$) were extracted from SliceTCA's $\boldsymbol{C}$ (**B.1**) and traces from SliceTCA's $\boldsymbol{v}$ (**B.2**). See details in Section H.2.

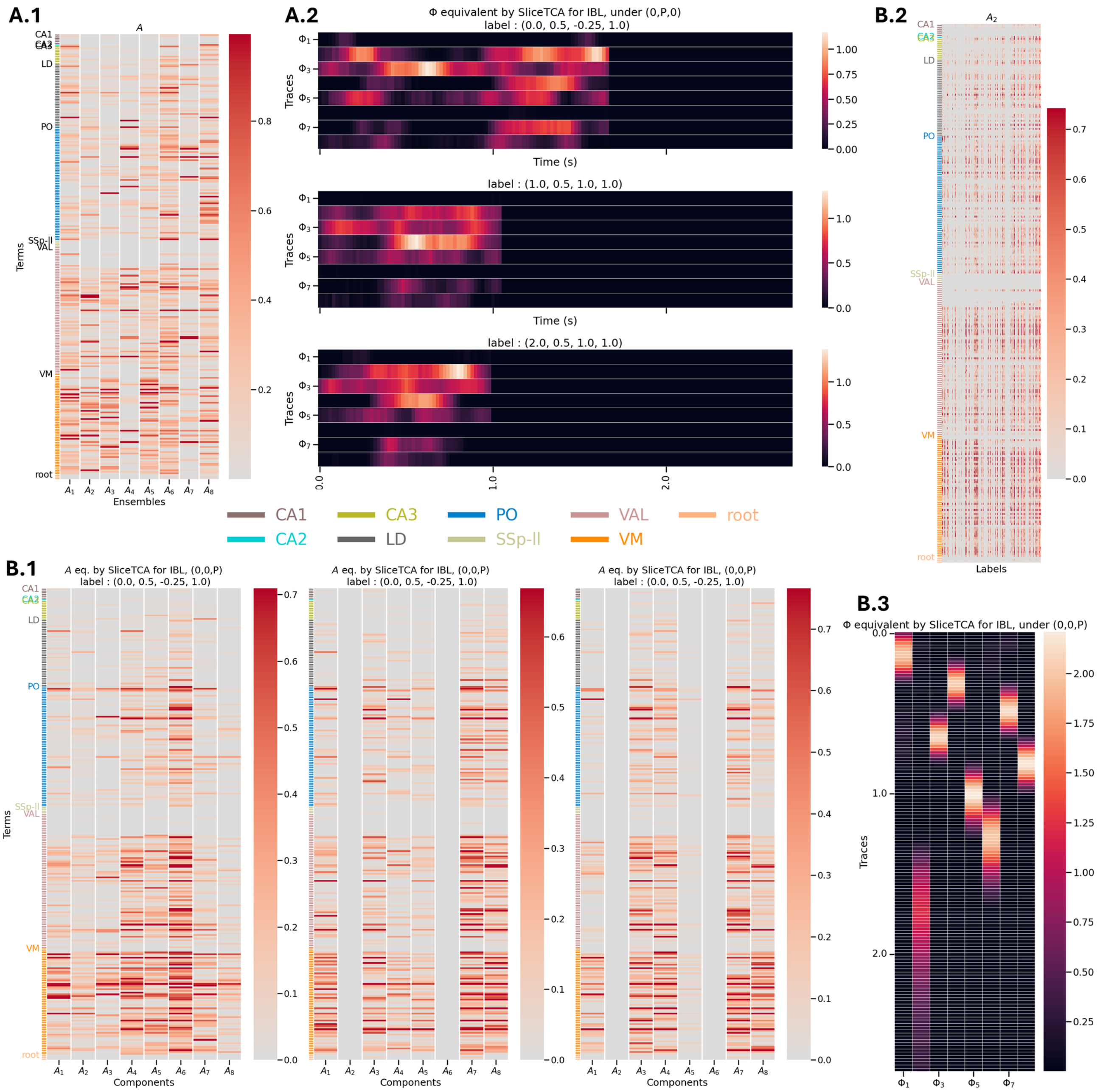

Figure 29. **IBL experiment: SliceTCA components. A:** sliceTCA's configuration 1 (see Sec. H.2). **A.1:** Components from the fixed component case (i.e., from sliceTCA's $\boldsymbol{u}$). **A.2:** Traces from the fixed component case (i.e., from sliceTCA's $\boldsymbol{A}$).
B: sliceTCA's configuration 2: **B.1:** Components from the varying component case. **B.2:** Components showing how they vary over labels (example: component 2). **B.3:** Traces from the sliceTCA's $\boldsymbol{v}$ vector. See details in Section H.2.

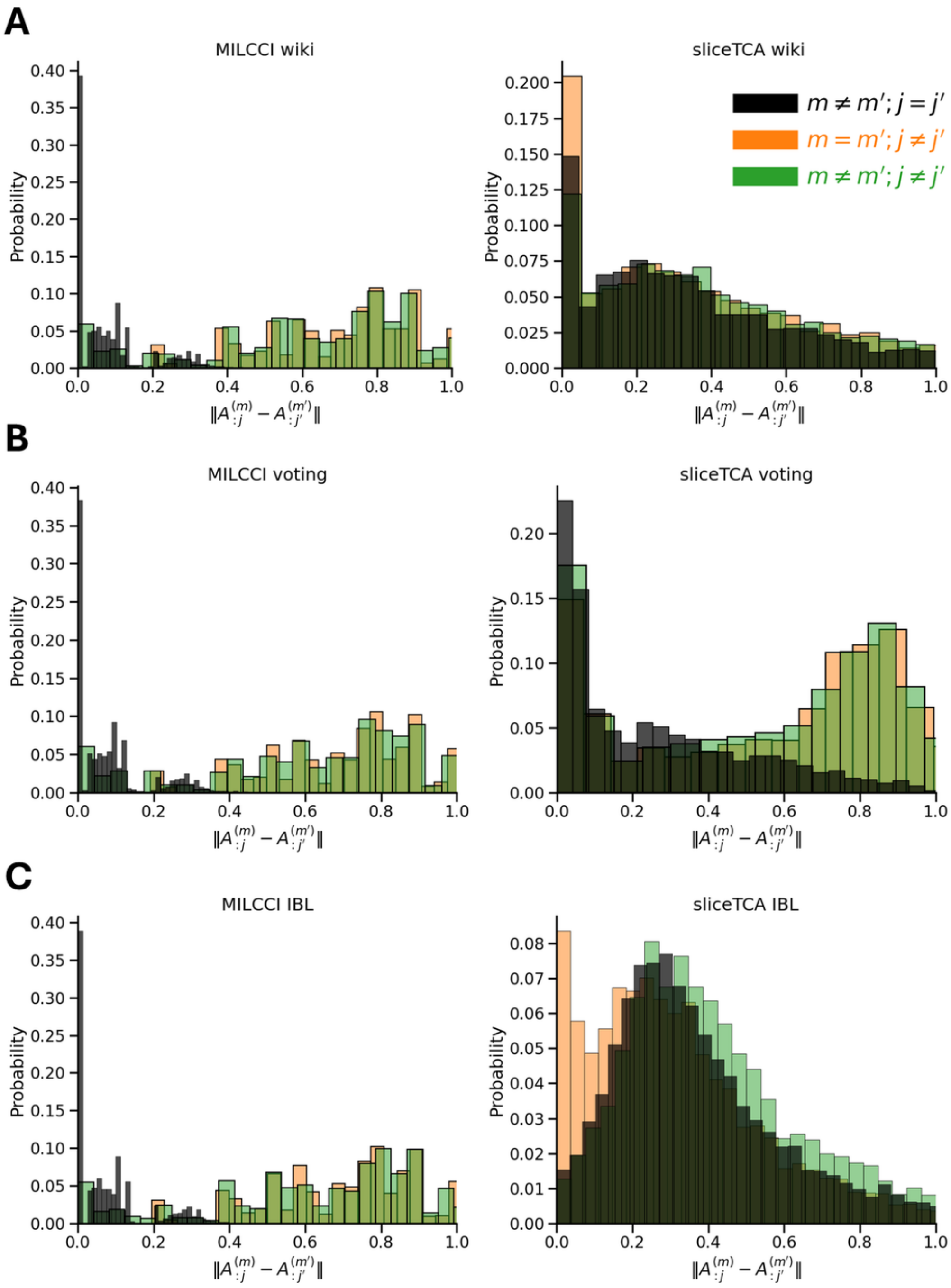

Figure 30. **Distribution of distances between component pairs.** Comparison of distances between same components (black) versus different component pairs across three datasets and two methods, presented for sliceTCA configuration 2 that allow components change (Sec. H.2). **A** Wiki dataset, **B** Voting dataset, **C** IBL dataset. Left column shows MILCCI results, right column shows sliceTCA results. Black bars represent distances between the same component across different conditions ($m \neq m'; j = j'$), orange bars represent distances between different components in the same condition ($m = m'; j \neq j'$), and green bars represent distances between different components in different conditions ($m \neq m'; j \neq j'$).

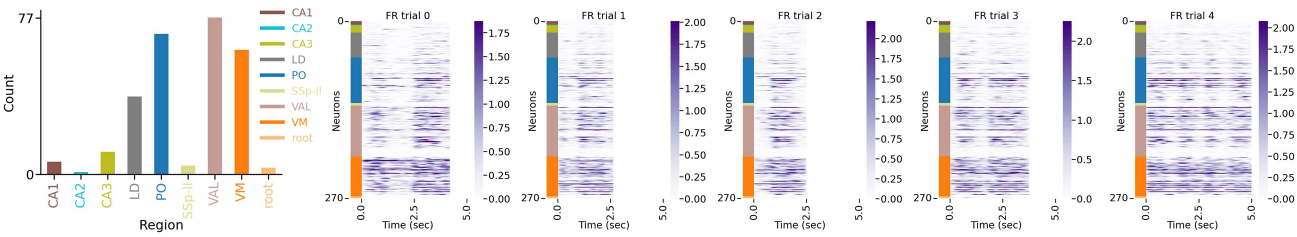

Figure 31. IBL data following our pre-processing steps.

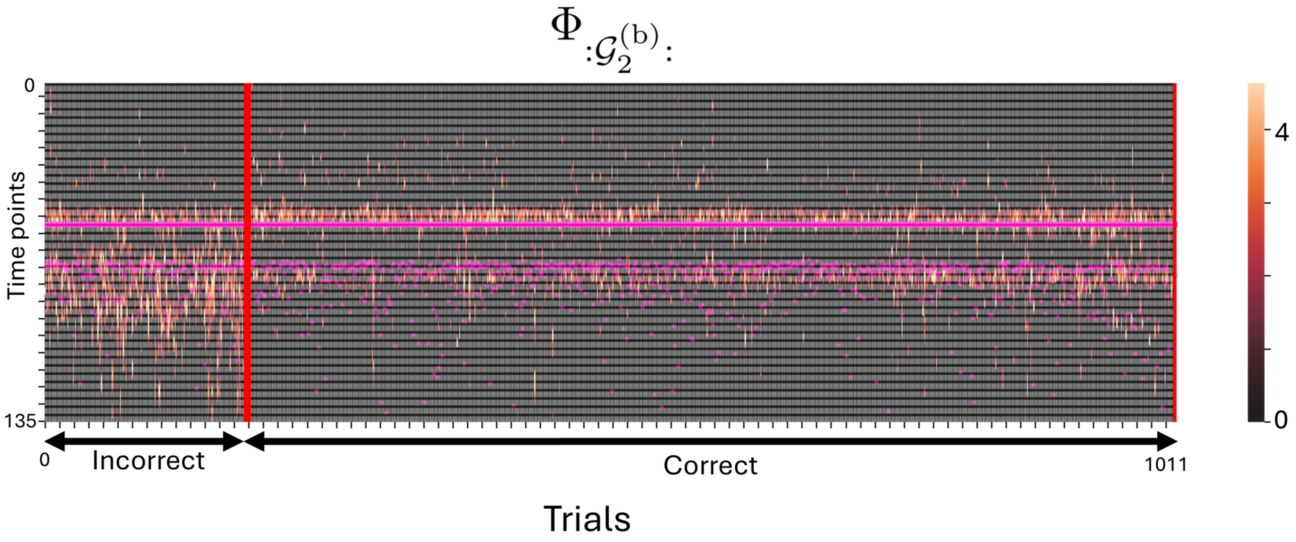

Figure 32. Traces of $\mathbf{\Phi}_{:\mathcal{G}_2^{(\mathrm{b})}:}$ across trials, separated by decision correctness. Solid pink: stimulus on. Dashed pink: stimulus off. The stimulus appears for a median duration of 1.45 s across trials.

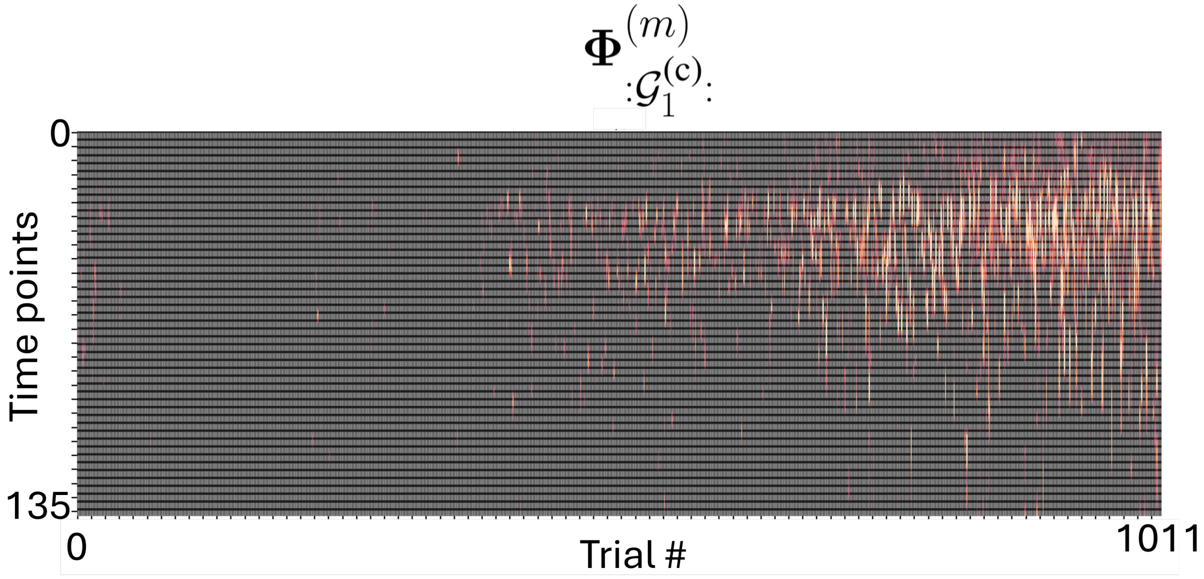

Figure 33. $\mathbf{\Phi}_1^{(\mathrm{c})}$ presents an increasing temporal drift over trials.

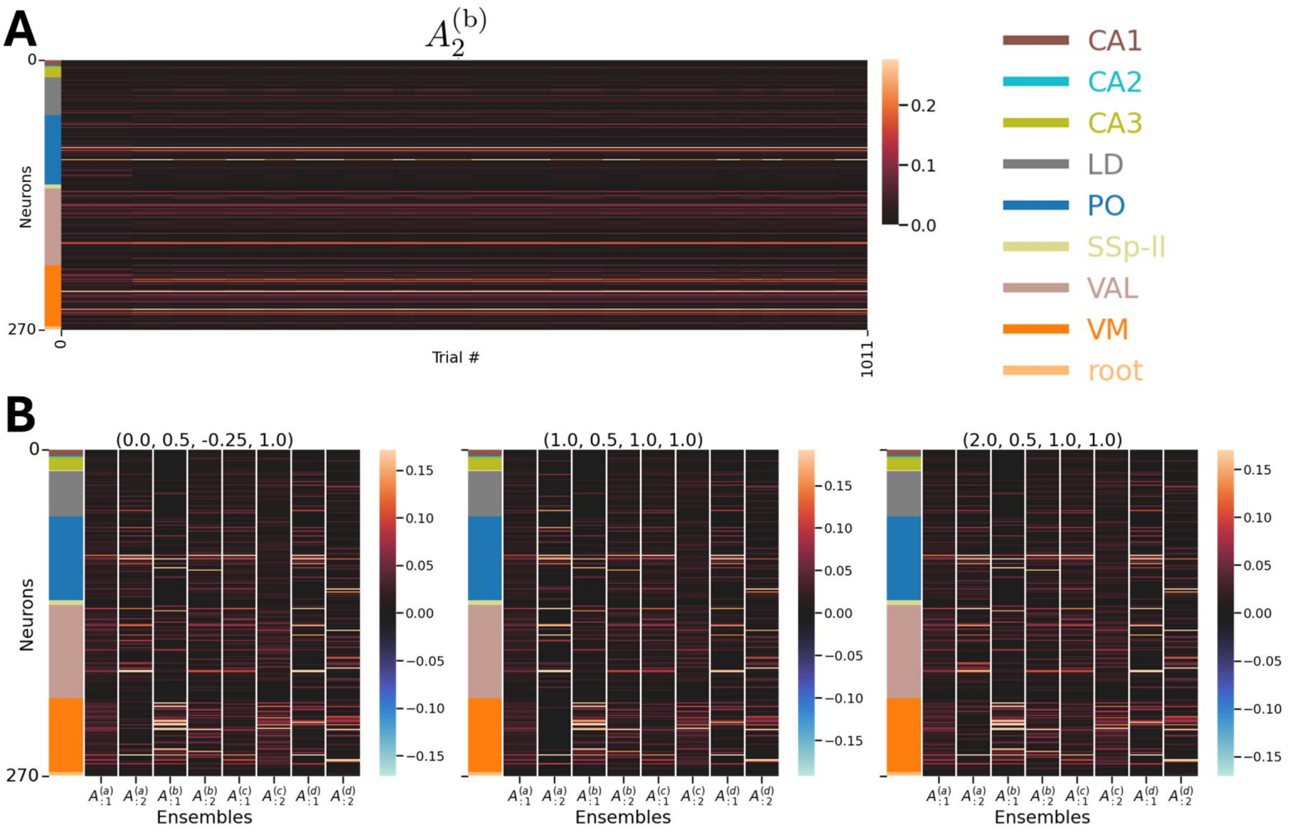

Figure 34. **Components identified by MILCCI in the IBL experiment. A:** Example ensemble (with its trace discussed in the main text) and its adjustments across trials. **B:** Example ensemble matrices reconstructed for three random trials. Each subplot shows all ensembles present in that trial under the unique set of labels indicated.

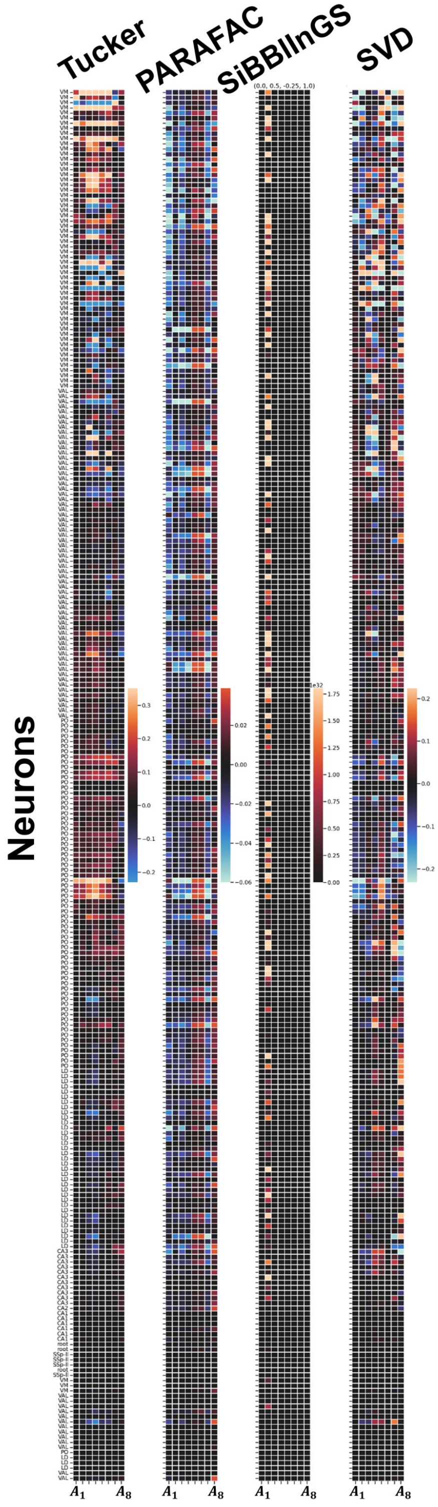

Figure 35. **IBL Neuronal Ensembles Components Identified by Baselines.**

G. Additional Information about Neuronal Ensembles Experiment

The IBL dataset is part of the International Brain Laboratory (IBL) effort to map neural activity underlying decision-making in mice across the whole brain. We accessed the IBL's data via the Dandi archive, in an NWB (Rübel et al., 2022; Laboratory et al., 2025) format.

The randomly selected session was recorded on February 11, 2020 in the Churchland Lab at CSHL (currently at UCLA). In the IBL task, mice view a grating stimulus on the left or right side of a screen (or no stimulus) and report its location by turning a wheel. The task includes block-wise priors, where one side is more likely than the other, requiring mice to combine sensory evidence with prior expectations. Stimulus contrast varies across trials to manipulate difficulty, enabling precise measurement of perceptual decision-making and neural correlates during electrophysiology recordings. Electrophysiological recordings were collected using Neuropixels probes from diverse brain areas. These recordings provide single-spike resolution activity during a decision-making task and include additional data such as sensory stimuli presented to the mouse, behavioral responses and response times. The subject (ID: CSHL052) was a female C57BL/6 mouse (Mus musculus), 6 months old and 22g at the time of recording. The full description of the session and task protocol is provided in (Angelaki et al., 2025).

H. Information About Baseline Calculation and Execution

We compared MILCCI to matrix (SVD), tensor (PARAFAC, HOSVD, sliceTCA (Pellegrino et al., 2024)), and multi-array (SiBBlInGS (Mudrik et al., 2024)) decompositions.

H.1. Comparison to SVD, Tucker, PARAFAC

We compared these methods to MILCCI both quantitatively and qualitatively. For the qualitative comparison, we provide example figures in the main text and appendix that emphasize MILCCI's superior performance. Notably, in all these methods the component matrix is fixed, as defined by the first mode; therefore, unlike MILCCI, they cannot (1) reveal structural adjustments over trials or disentangle category effects via the components, and (2) capture free trial-to-trial variability without tensor constraints (except for SVD). Consequently, their ability to capture such effects is inherently limited, though they represent the closest methods to MILCCI that we can reasonably compare to (in the sense that they provide comparable components and traces). Thus, the comparison is also limited in that we cannot show structural variability that these methods fundamentally do not support. Quantitatively, we calculated information criteria (AIC, BIC, HQC; lower values indicate better fit) using the degrees of freedom of each method. As seen in the appendix figures, these methods struggle to capture the data when constrained to the same dimensionality as MILCCI, which we attribute to (1) the need for small structural adjustments, and (2) their inability to capture free trial-to-trial variability.

See below running details for these methods:

- **SVD**: We used NumPy's 'linalg.svd' package, using the same number of components as in MILCCI for each experiment. The SVD was applied to the data from all trials concatenated horizontally.

  For experiments containing missing values (e.g., the voting experiment), NaNs were filled with zeros. Components were extracted from the left singular vectors (U), and the corresponding traces were obtained by multiplying the singular values matrix (Σ) with the right singular vectors ($V^\top$).

- **PARAFAC** (Harshman et al., 1970): We used the PyLops (Ravasi & Vasconcelos, 2020) PARAFAC implementation, with the same rank and number of components as MILCCI. For experiments with trials of varying durations (e.g., the IBL), we used the 90-th percentile trial length to prevent outliers from dominating, stacking trials along a third dimension and zero-padding shorter trials.

  Components ($\mathcal{A}$) were extracted from the first tensor mode (first factor), and traces were obtained by multiplying the second mode and the third mode according to the trial and component count.

- **Tucker** (Tucker, 1966) (HOSVD): Also for Tucker, we used the PyLops (Ravasi & Vasconcelos, 2020) PARAFAC implementation, with the same dimensions and number of components as MILCCI. For the 3-rd mode, we used the minimum between the number of time points and the number of trials. Again, for experiments with trials of varying durations (e.g., the IBL), we used the 90-th percentile trial length to prevent outliers from dominating, stacking trials along a third dimension and zero-padding shorter trials.

Components ($\mathcal{A}$) were extracted from the first tensor mode (first factor), and traces were obtained by multiplying the second mode, the core matrix, and the trial and component count. Notably, the component matrix in these methods is fixed, as defined by the core tensor, and therefore cannot adjust over time or disentangle label variability via the components.

For some datasets (e.g., Wikipedia), PARAFAC and Tucker (PyLops (Ravasi & Vasconcelos, 2020) implementation) could not converge at the same MILCCI dimensionality ($p = 12$), even with SVD initialization, various normalization schemes, high tolerance (1e-2), $\ell_2 2$ regularization, and maximum iterations of 10,000, due to least-squares optimization instability. These errors often occur due to (1) many missing values (though no NaNs exist in our data), or (2) high-resolution (daily) measurements introducing highly correlated (Fig. 27) or nearly linearly dependent structures in the data. Hence, for the Wikipedia comparison, in addition to comparisons to SVD, SiBBlInGS, and sliceTCA, we tested Tucker and PARAFAC under lower ranks with increasing added noise. We found that these models converge at rank 9 with added i.i.d. Gaussian noise ($\sigma = 0.1$ for Tucker and $\sigma = 0.2$ for PARAFAC), and the results presented here are under these conditions.

H.2. Comparison to SliceTCA

We note that a direct comparison between our method and SliceTCA (Pellegrino et al., 2024) is limited since SliceTCA is not tailored to find subtle label-driven supervised reorganization patterns in neural ensembles. SliceTCA performs an unsupervised decomposition $\hat{\boldsymbol{X}}_{n,t,k} = \sum_{r=1}^{R_{\text{neuron}}} u_n^{(r)} A_{t,k}^{(r)} + \sum_{r=1}^{R_{\text{time}}} v_t^{(r)} B_{n,k}^{(r)} + \sum_{r=1}^{R_{\text{trial}}} w_k^{(r)} C_{n,t}^{(r)}$ that finds R components. In their notation, $\boldsymbol{u}^{(r)}$ (neural loading vector) is equivalent to one column of our $\mathcal{A}$ (i.e., $\mathcal{A}_{:,j}$), while their slice $\boldsymbol{A}^{(r)}$ (time-by-trial matrix) would correspond to our $\boldsymbol{\Phi}$ for all trials. Similarly, $\boldsymbol{v}^{(r)}$ (time loading vector) combined with $\boldsymbol{B}^{(r)}$ (neuron-by-trial slice) provides an alternative decomposition. SliceTCA's R_{neuron} corresponds to our P.

We compared MILCCI to SliceTCA (Pellegrino et al., 2024) with $R_{\text{neuron}} = P$ using their publicly available implementation at `https://github.com/arthur-pe/slicetca`. We followed the parameters outlined in their Google Colab notebook: positive=True, learning_rate=5×10^{-3}, min_std=10^{-5}, max_iter=1,000 for Wiki and Synth, 5,000 for IBL, and seed=0.

We tested two separate configurations as baselines:

1. **Configuration (1):**
   considers one neural component (e.g., one ensemble) that varies its traces over trials without additions, which is the closest to MILCCI in terms of formulation, but not enabling small changes in ensembles.
2. **Configuration (2):**
   uses a matrix of neurons $\times$ trials (i.e., captures how neurons can change over trials) via the matrix $\boldsymbol{B}$, however each matrix of neurons by trial has one trace. This captures mainly the ensemble adjustment to trial in an unconstrained way (unlike MILCCI) and enables more flexibility in that, but on the other hand restricts the traces more. Any other combination of ranks would not be interpretable in terms of comparison to MILCCI.

We extracted MILCCI-equivalent $\{\mathcal{A}\}$ and $\boldsymbol{\Phi}$ as follows:

1. **Configuration (1)**:
   The $\boldsymbol{u}^{(r)}$ vectors form the ensemble matrix (fixed across trials), while temporal traces are extracted from the $\boldsymbol{A}^{(r)}$ matrices by breaking to trials.
2. **Configuration (2)**:
   The ensembles are captured by the rows of $\boldsymbol{B}^{(r)}$ and their variation over trials by the columns. Traces are given by $\boldsymbol{v}^{(r)}$.

H.3. SiBBlInGS

:

MILCCI's main advantage over SiBBlInGS (Mudrik et al., 2024) is interpretability for multi-way, multi-label data. Particularly, SiBBlInGS cannot disentangle the effects of co- or separately-varying labels, making it difficult to understand

their individual contributions. SiBBlInGS, by modeling each unique label tuple as a distinct label would further increase tensor size and computational complexity. This is especially pronounced in experiments where some label categories vary across many unique values (e.g., IBL trial number with over 1000 values). In contrast, MILCCI can handle all of these without requiring additional dimensions, and can also account for the ordinal nature of each category, whereas SiBBlInGS requires choosing a single sorting across all categories. While we acknowledge quantitative metrics would also be valuable against SiBBlInGS, SiBBlInGS' graph-driven sparsity makes standard model comparison metrics (AIC, BIC) irrelevant. Particularly, SiBBlInGS inference includes a graph-based reweighting sparsity mechanism that hinders accurate estimation of degrees-of-freedom needed for information criteria calculations, making information criteria comparison intractable. Hence, we limited quantitative comparisons against SiBBlInGS to synthetic data (Fig. 2) and, for the real-world experiments, we focused on qualitative comparisons that emphasize MILCCI's interpretability advantages.

I. Alternative Inference of traces via Dynamic Prior

In the main text, we regularize the temporal traces $\mathbf{\Phi}^{(m)}$ using a smoothness penalty. Here, we present an alternative formulation where the temporal traces evolve according to a Linear Dynamical System (LDS), suitable for data with non-stationary dynamics.

I.1. Linear Dynamical System Prior

We assume that for each trial m, the temporal traces follow:

$$\boldsymbol{\phi}_t^{(m)} = \boldsymbol{W}^{(m)} \boldsymbol{\phi}_{t-1}^{(m)} + \boldsymbol{\eta}_t, \quad t = 2, \dots, T^{(m)} \tag{4}$$

where $\boldsymbol{W}^{(m)} \in \mathbb{R}^{P \times P}$ is a trial-specific transition matrix and $\boldsymbol{\eta}_t \sim \mathcal{N}(\mathbf{0}, \sigma^2 \boldsymbol{I})$.

I.2. Modified Objective and Inference

We modify the optimization to jointly learn traces and dynamics. The algorithm alternates between:

Step 1: Update $\mathcal{A}^{(k)}$ (for each category k)

Same as main text, using Equation 2.

Step 2: Update $\{\mathbf{\Phi}^{(m)}, \boldsymbol{W}^{(m)}\}$ (for each trial m)

Given a fixed loading matrix for a general trial m, $\tilde{\boldsymbol{A}}$, we perform inner iterations (3-5 times):

Step 2a: Update $\mathbf{\Phi}^{(m)}$ given $\boldsymbol{W}^{(m)}$

$$\hat{\mathbf{\Phi}}^{(m)} = \arg\min_{\mathbf{\Phi}^{(m)}} \left\| \boldsymbol{Y}^{(m)} - \tilde{\boldsymbol{A}}(\boldsymbol{L}^{(m)}) \mathbf{\Phi}^{(m)} \right\|_F^2 + \gamma_3 \sum_{t=2}^{T^{(m)}} \left\| \boldsymbol{\phi}_t^{(m)} - \boldsymbol{W}^{(m)} \boldsymbol{\phi}_{t-1}^{(m)} \right\|_2^2 + \gamma_4 \left\| (\boldsymbol{C} \odot (\mathbf{1} - \boldsymbol{I}_P)) \odot \boldsymbol{D} \right\|_{1,1} \tag{5}$$

Step 2b: Update $\boldsymbol{W}^{(m)}$ given $\mathbf{\Phi}^{(m)}$

With regularization $R(\boldsymbol{W}^{(m)}) = \|\boldsymbol{W}^{(m)} - \boldsymbol{I}\|_F^2$ to encourage stability:

$$\hat{\boldsymbol{W}}^{(m)} = \left(\sum_{t=2}^{T^{(m)}} \boldsymbol{\phi}_t^{(m)} (\boldsymbol{\phi}_{t-1}^{(m)})^T + \gamma_5 \boldsymbol{I} \right) \left(\sum_{t=2}^{T^{(m)}} \boldsymbol{\phi}_{t-1}^{(m)} (\boldsymbol{\phi}_{t-1}^{(m)})^T + \gamma_5 \boldsymbol{I} \right)^{-1} \tag{6}$$

The inner iterations stabilize both $\mathbf{\Phi}^{(m)}$ and $\boldsymbol{W}^{(m)}$ before updating $\mathcal{A}$, preventing noise amplification.

I.3. Initialization

1. Initialize $\mathbf{\Phi}^{(m)}$ using the original smoothness-based objective (Equation 3)

2. Initialize $\boldsymbol{W}^{(m)} = \left(\sum_t \boldsymbol{\phi}_{t,\text{init}}^{(m)}(\boldsymbol{\phi}_{t-1,\text{init}}^{(m)})^T\right)\left(\sum_t \boldsymbol{\phi}_{t-1,\text{init}}^{(m)}(\boldsymbol{\phi}_{t-1,\text{init}}^{(m)})^T\right)^{-1}$

This dynamic prior is suitable for cases that are assumed to be stationary and governed by a single LDS that does not change over time. Notably, real-world data is often non-stationary, and hence this dynamic prior would benefit from extensions in future work, such as learning dynamics like those exemplified in (Chen et al., 2024; Mudrik et al., 2025)).

Table 4. Timing results (in seconds) for different methods across the three main experiments. Dash (-): method did not converge for the same dimension.

Experiment	SVD	Tucker	parafac	parafac_scaled	SliceTCA	SiBBlInGS	MILCCI
Voting	20.01	20.12	20.26	20.35	27.58	27.19	28.16
Wiki	20.37	-	-	-	34.05	29.48	31.12
IBL	24.03	268.94	1056.20	1256.74	1000	1004.32	1000.32

J. Fourth real-world experiment: MILCCI identifies neural ensembles that adjust to arousal level and stimulation frequency and evolve via dynamical rules

J.1. Data and Pre-processing

We used one experimental session from (Papadopoulos et al., 2024) in NWB format from the DANDI archive (Rübel et al., 2022) (DANDI:000986), which the McCormick laboratory at the University of Oregon collected. The dataset contains recordings from mouse auditory cortex during passive exposure to auditory stimuli. The subject was a male mouse (Mus musculus), aged P79D from birth (see experimental illustration taken from (Papadopoulos et al., 2024) in Fig. 36A).

We processed spike trains from the NWB file (raster plot in Figure 36E) to estimate firing rates (FR) using Gaussian convolution with the following parameters: 5 ms time bins, 50 ms Gaussian window, and 5 ms σ (Fig. 36F). For this demonstration, we used the first 500 trials. The processed dataset thus contain 235 neurons across 15 unique experimental conditions that varied throughout the first 500 trials.

We leverage this example to demonstrate MILCCI's robustness and capacity for extensions, including: 1) pre-processing via non-linearity, 2) modifying the inference of Φ to evolve via dynamical priors, rather than via Eq. 3 (App. I for details), and 3) robustness to hyperparameters (App. J.3).

J.2. Demonstration of Extended MILCCI With Non-Linear Transformation and Dynamics Prior Over Traces Evolution

This experiment provides a glimpse of MILCCI's extensibility by showcasing two key advances: (1) dynamical evolution of neural ensembles, and (2) nonlinear transformation of FR via tanh (normalizing to range [-1, 1]). This serves as a proof of concept for modeling dynamics alongside MILCCI, paving the way for future extensions with more complex non-stationary dynamics (see App. I for details on dynamics inference).

We defined the following categories: (a) arousal level (based on binarized pupil diameter, Fig. 36C); and (b) stimulation frequency (distribution among the used trials in Fig. 36B).

We ran extended MILCCI with $p_j = 3$ ensembles per category.

Here, MILCCI reveals interpretable structure across multiple experimental variables (Fig. 37). The learned transition networks $\boldsymbol{W}^{(m)}$ (Fig. 37A) show condition-dependent ensemble interactions, with varying positive and negative coupling strengths across arousal levels and stimulation frequencies. The heatmap of all transition matrices across trials (Fig. 37B) reveals systematic organization aligned with both arousal and frequency conditions. The temporal traces (Fig. 37C) capture joint structure: when trials are sorted by arousal (left color bar), $\boldsymbol{\Phi}_2$ and $\boldsymbol{\Phi}_3$ show distinct activation patterns that align with arousal levels, while the stimulation frequency (top color bar) reveals additional frequency-specific structure within arousal groups. This is further supported by the averaged traces (Fig. 37D), where conditioning on specific frequencies

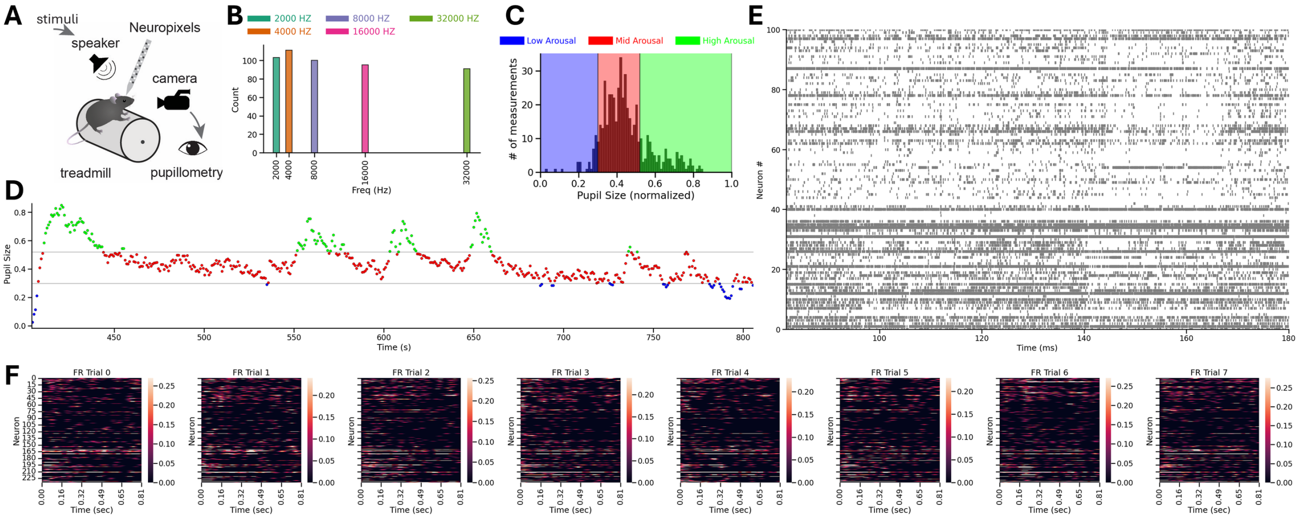

Figure 36. **Additional demonstration of MILCCI on data from (Papadopoulos et al., 2024). A:** Experiment illustration, adapted from (Papadopoulos et al., 2024). **B:** Trial counts per stimulation frequency among the first 500 trials analyzed. **C:** Distribution of normalized pupil size (a.u.) across the first 500 trials. Colored background represents the three arousal states considered. (cutoffs 0.3, 0.52) **D:** Average pupil size per trial. Each marker represents one trial; x-axis shows trial midpoint time from recording start. **E:** Raster plot of the first 180 ms of neural activity. **F:** Example firing rate estimation for the first 8 trials from spike train data.

(4, 8, 32 kHz) reveals distinct trace shapes that vary with arousal level. The identified ensembles (Fig. 37F) show subtle compositional adjustments to both arousal (top) and stimulus frequency (bottom), demonstrating MILCCI's ability to capture category-specific structural effects. The model achieves low reconstruction error across trials (Fig. 37E; mean relative MSE ≈ 0.07).

J.3. Hyperparameter Sensitivity Analysis

To empirically demonstrate MILCCI's robustness to hyperparameter choices, we conducted a comprehensive sensitivity analysis across a wide range of values. We tested 20 values for $\gamma_1 \in [0.002, 0.5]$ and 40 values for $\gamma_2 \in [0.002, 0.5]$, yielding 800 total hyperparameter combinations across multiple ensemble instances.

To quantify the differences of learned components $\mathcal{A}$ between iterations with different parameters vs. same parameters, we employed the normalized Frobenius distance:

$$d_{\text{norm}}(\widetilde{\mathcal{A}}^{(1)}, \widetilde{\mathcal{A}}^{(2)}) := \frac{\|\widetilde{\mathcal{A}}^{(1)} - \widetilde{\mathcal{A}}^{(2)}\|_F}{\sqrt{\|\widetilde{\mathcal{A}}^{(1)}\|_F^2 + \|\widetilde{\mathcal{A}}^{(2)}\|_F^2}} \tag{7}$$

where $\widetilde{\mathcal{A}}^{(1)}$ and $\widetilde{\mathcal{A}}^{(2)}$ are two general components for a pair of iterations. This metric enables us to compare the degree of change of the same ensemble under hyperparameter change to the degree of difference between 2 distinct ensembles. If a single ensemble remains more consistent under hyperparameter change compared to cross-ensemble differences, that means that the degree of hyperparameter sensitivity is slight, which suggests MILCCI's robustness.

We thereby computed two types of distances to assess hyperparameter sensitivity relative to ensemble variability (Fig. 38B,C,F):

- **Within-ensemble distances**: For each ensemble, we computed pairwise distances between component matrices obtained with different hyperparameter settings. These distances quantify how much the learned components vary due to hyperparameter choices while holding the data fixed.

- **Cross-ensemble distances**: For each hyperparameter setting, we computed pairwise distances between component matrices from different ensemble instances. These distances quantify how much the learned components vary due to data sampling and stochastic initialization while holding hyperparameters fixed.

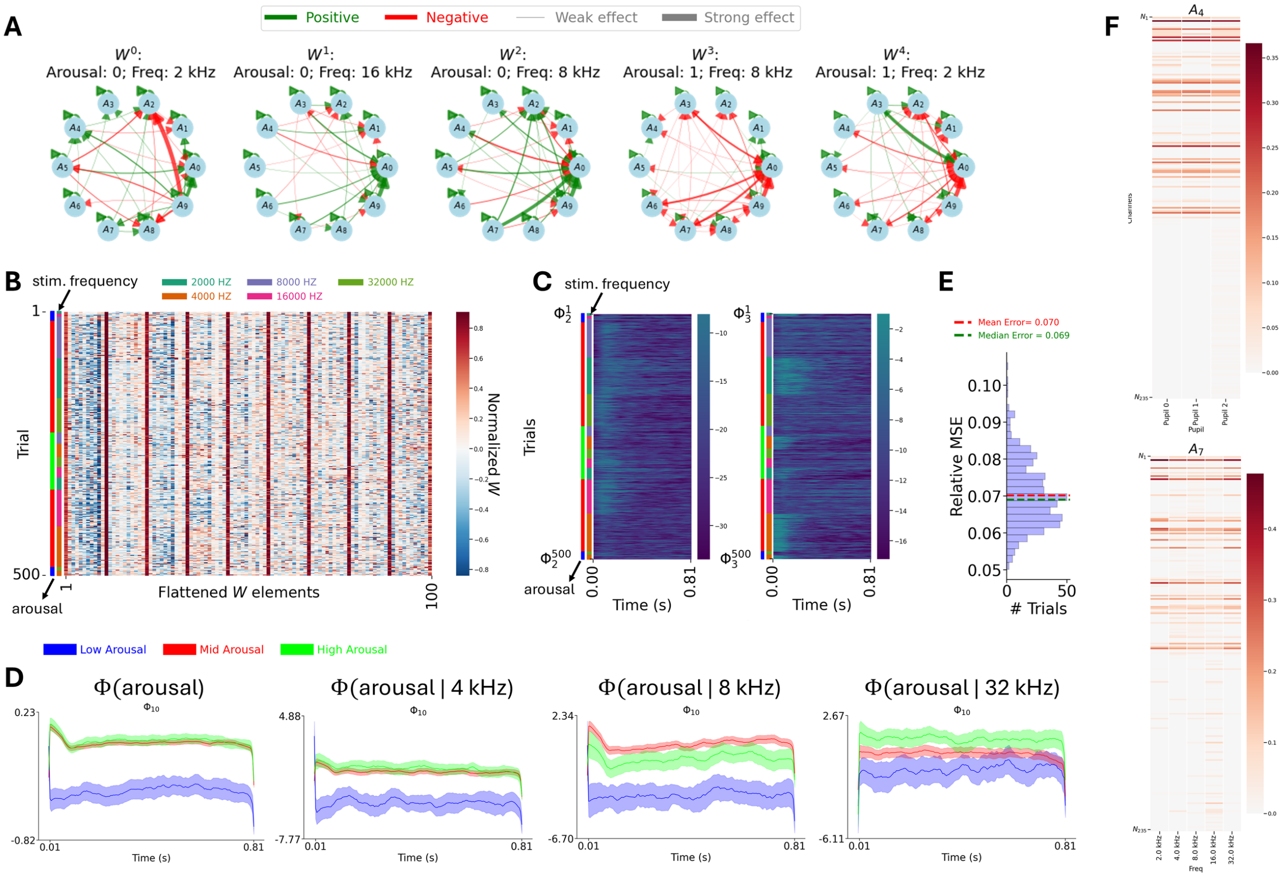

Figure 37. **Demonstrating MILCCI with nonlinear tanh data transformation** ($\tanh\left(\boldsymbol{y}^{(m)}\right) \approx \sum_{(\mathbf{k})\in C} \mathcal{A}^{(\mathbf{k})}_{::L^{(m)}_{\mathbf{k}}} \boldsymbol{\Phi}^{(m)}_{\mathcal{G}^{(\mathbf{k})}:}$) **and dynamical constraints over the trace evolution** ($\boldsymbol{\Phi}^{(m)}_t \approx \boldsymbol{W}^{(m)}\boldsymbol{\Phi}^{(m)}_{t-1}$) **, via recent neural data from (Papadopoulos et al., 2024). (A)** Transition networks $\boldsymbol{W}^{(m)}$ for five example trials from different arousal and stimulus frequency conditions, showing learned interactions between ensembles. **(B)** Heatmap of all learned transition matrices (each $\boldsymbol{W}^{(m)}$ flattened) across trials (rows). Scatters on the left indicate trial conditions: arousal level (left side) and stimulation frequency (right side). Weights are normalized by column maximum. **(C)** Temporal traces for two example ensembles ($\boldsymbol{\Phi}^1$ and $\boldsymbol{\Phi}^3$) across trials and time, organized by arousal level, demonstrating condition-dependent temporal dynamics. Scatters on the left are the same as in panel **B**. **(D)** Average temporal traces with stderr grouped by arousal level: without frequency conditioning (left), or conditioned on specific stimulus frequencies (three rightmost panels: 4 kHz, 8 kHz, and 32 kHz). **(E)** Distribution of reconstruction error (relative MSE) across trials. Dashed lines indicate mean and median. **(F)** Example neuronal ensembles and how they subtly adjust to labels, showing condition-specific loadings across channels. Top panels show components varying with arousal level; bottom panels show components adjusting to stimulus frequency.

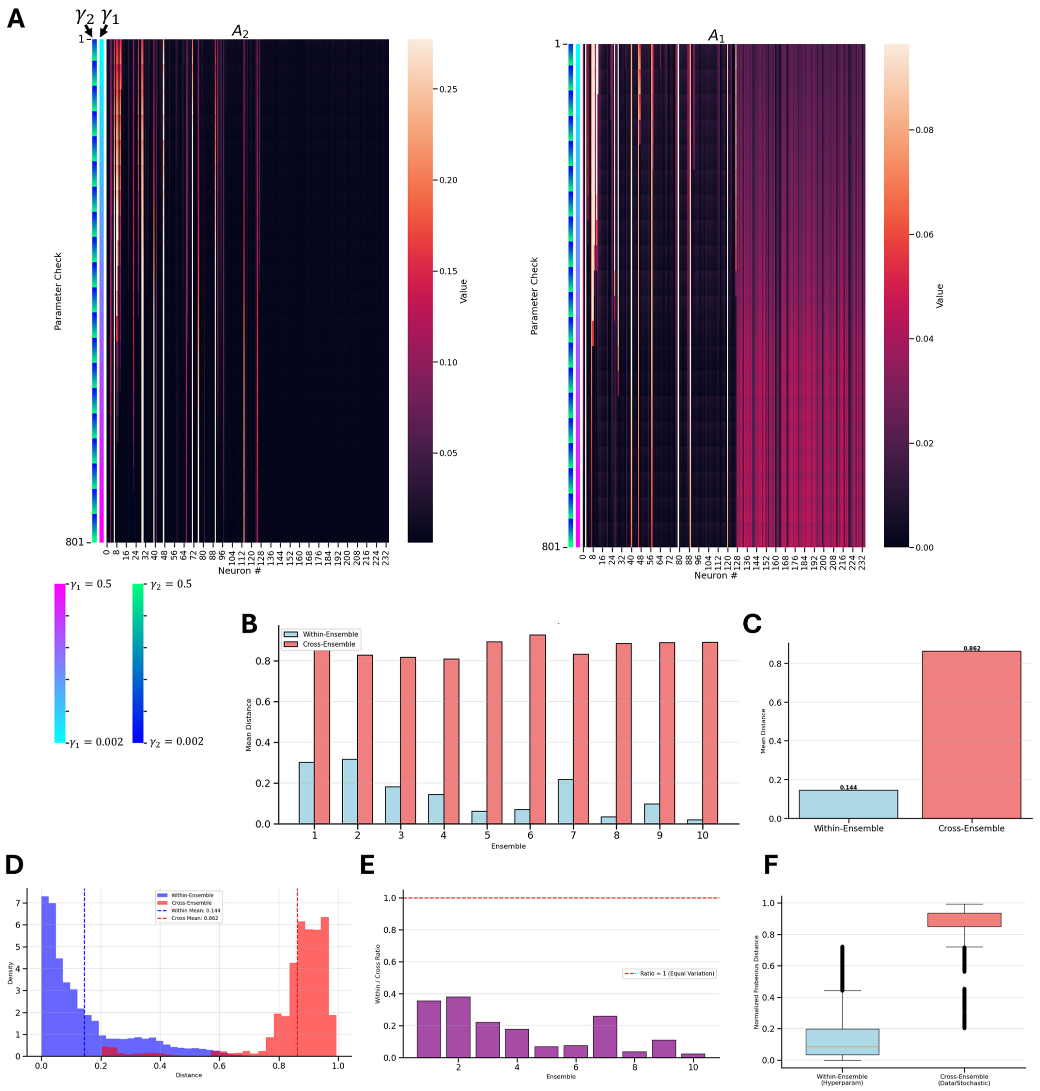

Figure 38. **Hyperparameter sensitivity analysis across 800 parameter combinations (App. J.3). A:** Example component matrices from two ensembles across all hyperparameter settings, ordered by γ_1 and γ_2 (colorbars at the bottom). For example, higher γ_1 values yield sparser components. **B:** Per-ensemble comparison of within-ensemble (blue) versus cross-ensemble (red) distances. **C:** Overall mean distances showing within-ensemble variation is smaller than cross-ensemble variation. **D:** Distribution of normalized Frobenius distances reveals clear separation between within-ensemble and cross-ensemble variation. **E:** Robustness ratios for each ensemble, all below 1 (dashed line indicates equal variation). **F:** Overall boxplot comparison confirms systematic difference between within-ensemble and cross-ensemble distances across all 10 ensembles.

As seen in Fig. 38, B-F, the within-ensemble distances are substantially smaller than cross-ensemble distances. This would indicate that the learned components are more sensitive to the underlying data structure than to hyperparameter tuning, demonstrating that the model reliably captures meaningful patterns across reasonable hyperparameter ranges.

Interestingly, the component matrices (Fig. 38A) show that higher γ_1 values produce sparser components as expected, while the overall structure remains stable. Within-ensemble distances are consistently lower than cross-ensemble distances, with a mean ratio of 0.17 (Fig. 38C), indicating that hyperparameter variation introduces less variability than ensemble stochasticity. The distribution of distances (Fig. 38D) reveals separation between the two types of variation. Per-ensemble analyses (Fig. 38B,E) show this pattern holds across all individual ensemble instances, with ratios below 1. These results demonstrate model robustness, as hyperparameter variation contributes minimally compared to stochastic ensemble variation.

K. Ethics Statement and LLM Usage

Our work does not raise any ethical concerns. Large language models were used only at the word or sentence level during manuscript writing to improve the language and catch grammar mistakes, with no influence on the scientific content or analysis.
